# “All-in-a-tube” detection of RDX and TNT: old silver mirror reaction revived for nitro-explosive quantification

**DOI:** 10.1007/s00604-025-07195-w

**Published:** 2025-05-08

**Authors:** Selen Durmazel, Ayşem Üzer, Reşat Apak

**Affiliations:** 1 Analytical Chemistry Division, Department of Chemistry, Faculty of Engineering, Istanbul University-Cerrahpaşa, Avcilar 34320, Istanbul, Turkey; 2 Turkish Academy of Sciences (TUBA), Çankaya, 06670 Ankara, Turkey

**Keywords:** Colorimetric detection, 1,3,5-Trinitroperhydro-1,3,5-triazine (RDX), 2,4,6-Trinitrotoluene (TNT), Tollens’ reagent, Silver nanoparticles

## Abstract

Inspired by analyte-induced in situ formation/growth of silver nanoparticles (AgNPs), the traditional Tollens’ reagent of diamminesilver(I) complex cation [Ag(NH_3_)_2_]^+^ was shown to be capable of detecting explosives. Thus, a color test based on in situ formation of AgNPs was developed for 1,3,5-trinitroperhydro-1,3,5-triazine (RDX) and 2,4,6-trinitrotoluene (TNT). The resultant yellow-colored AgNPs were characterized using UV–visible spectrophotometry, scanning transmission electron microscopy, and dynamic light scattering measurements. The system displays turn-on absorbance behavior with RDX and TNT, yielding detection limits of 50.3 and 67.2 nmol L^−1^, respectively. The assay could distinguish between RDX and TNT using a simple extraction-based recovery procedure. Good recoveries for RDX and TNT were obtained from real (Composite B, Composite A5, and Octol) and synthetically prepared formulations. Method validation was performed by statistically comparing the analytical results obtained by the reference liquid chromatography-tandem mass spectrometric method applied to RDX standards and RDX-contaminated soil samples. As a novelty of this system, direct spectrophotometric detection of RDX through its decomposition product, formaldehyde, was successfully performed for the first time. Compared with the widely used indirect spectrophotometric methods based on nitrite formation from RDX degradation, the system is superior in that it does not require pre-hydrolysis of RDX and does not respond to 1,3,5,7-tetranitro-1,3,5,7-tetraazacyclooctane (HMX). It is also noteworthy that the system responds to TNT in a way that constitutes an innovative alternative to the existing mechanism of Meisenheimer/Janowsky complex formation in the literature.

**Graphical Abstract:**

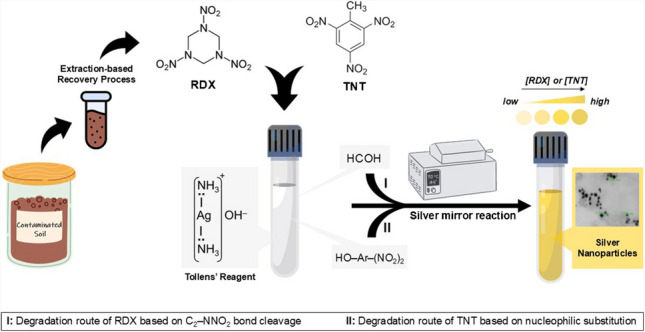

**Supplementary Information:**

The online version contains supplementary material available at 10.1007/s00604-025-07195-w.

## Introduction

The best-known nitramine- and nitroaromatic-type energetic materials (EMs), 1,3,5-trinitroperhydro-1,3,5-triazine (Royal Demolition eXplosive, RDX) and 2,4,6-trinitrotoluene (TNT), respectively, were found sufficient for all weapons applications in the past. However, new munition formulations emerged in line with the needs to increase explosion performance and/or ensure safety in ammunition depots [1–3]. Munition formulations basically consist of binary or ternary mixtures of these EMs or their coated forms with polymeric/plastic materials to increase their storage and transportation stability [4, 5]. Some selected munition formulations based on the sensitive high EMs RDX and/or TNT, especially Composite B (60% RDX + 39% TNT + 1% wax) make it important to determine the main components of these formulations. Fast and accurate detection of the active compound or precursors/degradation products of EMs is an indispensable research topic for military and criminologic laboratories personnel for investigating post-blast sites, law enforcement modeling in anti-terrorism activities, and also environmental monitoring of remediation sites [6]. One of the low-cost techniques commonly used for on-site/in-field analysis is colorimetry [7]. Simple color tests for the detection of nitroaromatic- and nitramine-type compounds in pre-blast material or post-blast residue were first developed under the leadership of Jenkins and Walsh [2]. Subsequently, in 2008, Jiang et al*.* brought a new breath to this field, and for the first time, an energetic substance (TNT) was determined at the picomolar level by a relatively new combination, a nanoparticle (NP)-based colorimetric method [8]. As is known, these materials, especially noble metal NPs, have significant physico-chemical advantages, such as increased surface area, particularly strong surface plasmon resonance absorption of gold (Au) and silver (Ag) NPs, and superior enzyme-like catalytic activity properties [7]. The strong absorption of noble metal NPs in the visible region has become an indispensable sensing tool for naked-eye detection platforms. The general signal generation principles/mechanisms of noble metal NPs, briefly classified as predominantly aggregation/anti-aggregation, formation/growth, ligation/dissociation/displacement, and enzyme-like catalytic activity, have also taken their place in the detection of energetic substances with the naked eye.

Compared to the charge-transfer based detection of TNT bearing strong electron-withdrawing groups (−NO_2_) in the benzene ring that can form acceptor–donor interactions with Lewis bases, RDX is devoid of such groups and also lacks typical absorption bands derived from the π−π* or n−π* transitions; thus, only a few conventional and nanomaterial-based colorimetric assays have been reported to detect RDX.

The sensing principles of conventional colorimetric methods [7] applicable to TNT and RDX are based on the following:

- Chemical reduction of nitro (−NO_2_) to amino (−NH_2_) groups [9] or stacking complexes through π–π bonding with an electron-rich chromophore [10–12] for TNT
- Alkaline or photo-induced pre-degradation/reduction to nitrite ions or ammonia/amine species and subsequent use of a Griess [2] or Berthelot [13] type reaction, selective for the degradation products of RDX

The sensing principles of noble metal nanomaterial–based colorimetric methods applicable to TNT and RDX are based on the following:

- Monocolorimetric spectral variation depending on the distance between particles: color change that usually occurs as a result of aggregation [8, 14–16] or anti-aggregation [17, 18] for TNT
- Mono/multicolorimetric spectral variation depending on morphology/size: color change usually resulting from target-controlled etching of Ag nanoprisms [19] or AuNP/Griess–based mechanism after alkaline pre-degradation process [20] for RDX

Considering all the aforementioned sensing mechanisms, chemical/photolytic cleavage of the –N−NO_2_ bond of hexyl nitramine functional sites to yield NO_2_^−^ from RDX and Meisenheimer complexation from TNT have been mainly and successfully exploited by many researchers for conventional and nanomaterial-based methods, indirectly for RDX [21, 22]. However, there is almost no direct colorimetric method for recognizing RDX among structurally similar compounds. In addition, explosives detection based on the formation of noble metal NPs is not yet available in the literature because of their low redox activities. Inspired by the studies based on formaldehyde-induced formation of Ag nanomaterials via Tollens’ reagent (TR): [Ag(NH_3_)_2_]^+^ [23–25], we have put forward a nanoparticle formation–based colorimetric approach for the first time in this work to directly detect RDX and also TNT.

## Experimental

### Materials, chemicals, and instrumentation

The energetic materials used throughout the study, RDX (containing 85% active matter), TNT (pure), 2,4,6-trinitrophenylmethylnitramine (tetryl, pure), 2,4,6-trinitrophenol (TNP, pure), 2-amino-4,6-dinitrotoluene (2A-DNT, pure), 4-amino-2,6-dinitrotoluene (4A-DNT, pure), 1,3,5,7-tetranitro-1,3,5,7-tetraazacyclooctane (HMX, pure), pentaerythritol tetranitrate (PETN, pure), Composite B (containing 60% RDX, 39% TNT, and 1% wax), Octol (containing 70% HMX and 30% TNT), and Composite A5 (containing 99% RDX and 1% filler material) were kindly provided in low amounts by the Mechanical and Chemical Industry Corporation of Türkiye (Makine Kimya Endüstrisi Kurumu-MKEK; Ankara, Türkiye) from previous projects.

All reagents were analytical grade and used without further purification. All materials and chemicals, as well as equipment used throughout the study are given in the Supplementary Information.

### Preparation of solutions

Each solution used throughout the study is prepared as described in the Supplementary Information.

### Recommended “all-in-a-tube” procedure for RDX and/or TNT direct determination

To prepare the required reaction mixture for simultaneous hydrolytic degradation and colorimetric determination of RDX and/or TNT, 0.60 mL of 1.80 × 10^−3^ mol L^−1^ AgNO_3_ solution, 0.40 mL of 0.08 mol L^−1^ NaOH (waited for approximately 30 s for the formation of Ag_2_O precipitation), and 1.0 mL of 2.0 × 10^−2^ mol L^−1^ aq. NH_3_ were added to a test tube, respectively. Then, $x$ mL of RDX and/or TNT standard or soil-extract solution and $(3.0-x)$ mL of ultrapure water was introduced into the test tube, and the reaction mixture was incubated for 45 min in a thermostated water bath at 70.0 °C for the hydrolysis of RDX and/or TNT followed by the in situ formation of AgNPs. At the end of this time, the test tube was cooled to room temperature, and a pale yellow silver sol formation was observed followed by an increase in absorbance which was directly proportional to analyte concentration. The LSPR absorbance maximum at 400 nm was recorded against a blank solution (excluding RDX and/or TNT) using a UV/vis spectrophotometer. Application of the recommended procedure to RDX and/or TNT samples was schematized in Scheme S1.

### Colorimetric tests for elucidation of the sensing mechanism

In order to elucidate the alkaline degradation products of RDX and TNT under optimized conditions of the assay and to judge whether these degradation products contribute to the working principle of the method, the Purpald® assay [26] for detecting formaldehyde, the conventional Griess assay [27] for testing nitrite, and the main cupric reducing antioxidant capacity (CUPRAC) method [28] for testing phenolics were applied, as described in the Supplementary Information.

### Real sample analysis

As a certified reference material, clean sandy loam (CLN SOIL-3) was prepared for use in real sample analysis by contaminating it with the relevant analytes in varying combinations. For this purpose, 1 g of weighed soil sample was transferred to a series of beakers. Then, it was contaminated by adding 2.50 mL of 320 µg mL^−1^ RDX, 320 µg mL^−1^ TNT, and 640 µg mL^−1^ RDX:TNT (1:1, w/w) solutions separately. For the blank sample solution, the soil sample weighed as described above was contaminated with 2.50 mL of acetone and used as the blank sample solution in order to evaluate the results more accurately. The contaminated soil samples were homogenized by ultrasonication and dried in a vacuum oven at 50 °C for 1 h.

### Extraction-based recovery process of RDX and TNT

RDX and TNT were separated through an extraction-based recovery process. The method was applied as described above to each of the solutions obtained from the recommended extraction procedure detailed in the Supplementary Information. Absorbance measurements were made against a blank sample solution with a UV–visible spectrophotometer, and visible spectra were collected. Using absorbance values at a 400-nm wavelength, recoveries (%) of RDX and TNT were calculated. Application of the recommended extraction-based recovery process to contaminated soil samples was schematized in Scheme S2.

### Analysis of synthetic and real munition formulations

First, the proposed procedure was applied to a series of synthetic mixtures containing varying mass ratios of RDX and TNT, and the system was evaluated for absorbance additivity. On the other hand, each synthetic (amatol and pentolite as TNT-based munition formulations) and real munition formulations as Comp B, Comp A5, and Octol as military-purpose explosive mixtures used throughout the study were also prepared and analyzed. Using absorbance values at a 400-nm wavelength, RDX and/or TNT recovery values were calculated in percentage ratios (%).

### Possible ınterference effects on RDX determination

The proposed method was applied to certain energetic materials at a ten-fold molar ratio of RDX, namely, TNT (as the other analyte), HMX, TNP, tetryl, 2A-DNT, 4A-DNT, and PETN; soil components such as humic acid; common soil ions at a ten-fold molar ratio of RDX, that is, Cl^−^, NO_3_^−^, SO_4_^2−^, CO_3_^2−^, Mg^2+^, Ca^2+^, Cu^II^, Fe^II/III^, and possible camouflage materials at varying molar ratios (such as one-, five-, ten-fold of RDX), specifically sugar {D-(+)-glucose as a representative compound}, acetylsalicylic acid-based analgesic (aspirin) along with 0.64 mg L⁻^1^ of RDX (final conc.). The interference effect of Cu^II^ and D-(+)-glucose on RDX determination was eliminated by solubility differences of the species in acetone as a solvent. Then, the selectivity of the method was evaluated, and the recovery values (%) of RDX were calculated.

### LC−MS/MS conditions for RDX determination

The reference LC–MS/MS method [29] existing in the literature was applied to RDX detection with some modifications, and the analyses of RDX samples were done using the apparatus described in one of the previous studies of our research group [30]. Experimental details of the reference LC–MS/MS method are as described in the Supporting Information. For method validation, simultaneously obtained findings of the recommended (spectrophotometric) and reference (LC–MS/MS) methods were statistically compared.

### Statistical analysis

Statistical evaluation of analytical results was performed using Excel software (Microsoft Office 2013) for calculating the mean and standard deviation. Results were expressed as the (mean ± standard deviation (SD)). Method validation against both the literature LC−MS/MS determination route of RDX was made by means of Student’s *t-* and *F-*tests.

## Results and discussion

### Designing and optimizing the Tollens’ reagent (TR)-driven sensing system

The biggest inspiration of our work was the numerous literature studies reported for in situ AgNPs formation/growth-based determination of formaldehyde [23–25, 31–33], one of the primary alkaline degradation products of RDX [34]. Thus, we adapted the TR consisting of AgNO_3_, NaOH, and aqueous ammonia (aq. NH_3_) components to the simultaneous in situ hydrolysis of RDX and its direct determination, which we called the TR-driven “all-in-a-tube” system. It is known that the alkaline hydrolytic degradation of the target analyte RDX yields formaldehyde as an end product [34], whereas formaldehyde is not among the alkaline degradation products of TNT [35]. Since the degradation kinetics of RDX in an alkaline medium becomes faster with temperature and the stability of the end product formaldehyde is critical under these conditions (this is not the case with TNT), optimization studies were conducted with RDX as a representative analyte.

Some critical parameters were examined using both low and high concentrations (1.50 and 2.50 mg L^–1^ in final conc., respectively) of the representative analyte, RDX. In this direction, optimization studies were initiated by specifying the amount of Ag^+^. As shown in Figure S1, experiments were carried out in the initial concentration range of 1.60–2.0 mmol L^−1^; the appropriate final concentration of AgNO_3_ was determined as 2.16 × 10^−4^ mol L^−1^. The optimal amount of NaOH is significant for both diammine Ag(I) complex formation and high-yield alkaline hydrolysis of RDX at low and high concentrations. During the experiments carried out with NaOH in the concentration range of 0.01–0.20 mol L^−1^, the analytical signal started to decrease significantly from the NaOH concentration of 0.10 mol L^−1^. A critical decrease in LSPR band intensities and red-shift of the peaks due to the formation of an ultrathin Ag_2_O layer on the surface of AgNPs under extreme alkalinity and oxidative conditions was reported by Yin et al. [36]. Also, under strongly alkaline conditions (pH ≥ 12) with increasing temperature, aldehydes are likely to undergo aldol condensation and the Cannizzaro reaction [34]. The possibility that formaldehyde (i.e., the degradation product of RDX assumed to serve the detection mechanism) is transformed into formate and nitrate as a result of the abovementioned side reactions under strongly alkaline conditions is expected to consume the amount of free HCHO, explaining the sudden decrease in the optimization graph in the presence of NaOH at ≥ 0.10 mol L^−1^ concentrations. For this reason, selection of the best NaOH concentration was made by comparing the analytical signals obtained for RDX at both low and high concentrations with NaOH in the initial concentration range of 0.01–0.09 mol L^−1^. Accordingly, the appropriate NaOH concentration of 6.40 × 10^−3^ mol L^−1^ (final conc.) was chosen to yield the maximal analytical signal obtained for both concentrations of RDX, shown as 0.08 mol L^−1^ (initial conc.) in Figure S2. Choosing the appropriate concentration of aq. NH_3_ is effective in identifying the complex stoichiometry. In addition, excessive aq. NH_3_ should be avoided as it makes the test much less sensitive, leading to a reduction in the amount of free formaldehyde [37]. In experiments carried out with aq. NH_3_ within an initial concentration range of 12–20 mmol L^−1^ at the optimal AgNO_3_ concentration, the Ag_2_O precipitate formed (Eq. (1)) in alkaline medium at aq. NH_3_ concentration below 18 mmol L^−1^ could not be dissolved as [Ag(NH_3_)_2_]^+^ complex. The experiments were continued with 20 mmol L^−1^ aqueous ammonia to guarantee the solubility of the Ag_2_O precipitate (Eq. (2)).

1 $$2{\text{AgNO}}_{3(\text{aq})}+2{\text{NaOH}}\begin{aligned}\rightleftharpoons\;\end{aligned} {\text{Ag}}_{2}{\text{O}}_{(\text{s})}+2{\text{NaNO}}_{3}+{\text{H}}_{2}\text{O}$$

2 $${\text{Ag}}_{2}{\text{O}}_{\left(\text{s}\right)}+4{\text{NH}}_{3}+2{\text{NaNO}}_{3}+{\text{H}}_{2}\text{O}\begin{aligned}\rightleftharpoons\;\end{aligned}2\left[\text{A}{\text{g}({\text{NH}}_{3})}_{2}\right]{\text{NO}}_{3}+2\text{NaOH}$$

The species distribution diagram of the Ag(I)-NH_3_ complex system formed by the reactions in the presence of the specified amount of Ag^+^ and aq. NH_3_ was constructed taking into account the amount of free ammonia present in the medium. According to the species distribution diagram shown in Figure S3, initially all of the silver is present as Ag^+^ at very low aq. NH_3_ concentrations. As the aq. NH_3_ concentration increases, the initially formed 1:1 complex makes a negligible contribution to the system, while the dominant species soon becomes the 1:2-coordinated [Ag(NH_3_)_2_]^+^ ion [38]. Silver(I) is predominantly present as the two ligand-complexed ion even at free aq. NH_3_ concentrations as low as 10^–2.2^ M [39]. Accordingly, it is clear that under the favorable experimental conditions of the proposed procedure, there is an excess of the ligand (NH_3_) and all Ag^+^ ions in the system exist as the Ag(I)-diammine complex cation. This reduces the standard redox potential of the Ag^+^/Ag pair from 0.80 to 0.38 V for the Ag(NH_3_)_2_^+^/Ag^0^ pair. Thus, AgNPs formation can be realized in a kinetically faster and size-controlled manner.

In the last step of the optimization studies, experiments for choosing the incubation temperature and time in the range of 45–70 °C and 5–60 min, respectively, were performed for both low and high concentrations of RDX. Maximal analytical signals were obtained after incubation at 70 °C for 45 min, as seen in Figures S4 & S5, respectively.

### Plausible sensing mechanism and characterization studies

After preliminary and optimization studies, the conventional Tollens’ test [37] has enabled the TR-driven “all-in-tube” colorimetric detection of RDX through its degradation products with reducing properties in alkaline media. One of the main end products of alkaline hydrolysis (12 ≥ pH > 10) of RDX in aqueous systems is formaldehyde [34]. The oxidation of an aldehyde (HCOH) to carboxylic acid (HCOOH) involves the transfer of two electrons and requires two Ag (I) ions through a free radical intermediate by the mechanism shown in Figure S6. In light of this information, and also considering Eqs. (1) and (2), the overall reaction equation for the reduction of diamminesilver (I) ion with formaldehyde is shown in Eq. (3).

3 $$\begin{aligned}{2\text{Ag}{\text{NH}}_{3})}_{2}^{+}+\text{HCOH}+3{\text{H}}_{2}\text{O }\rightleftharpoons\;2{\text{Ag}}_{(s)}^{0}+4{\text{NH}}_{3}+\text{HCOOH}+{2{\text{H}}_{3}\text{O}}^{+}\end{aligned}$$

**Scheme 1:**
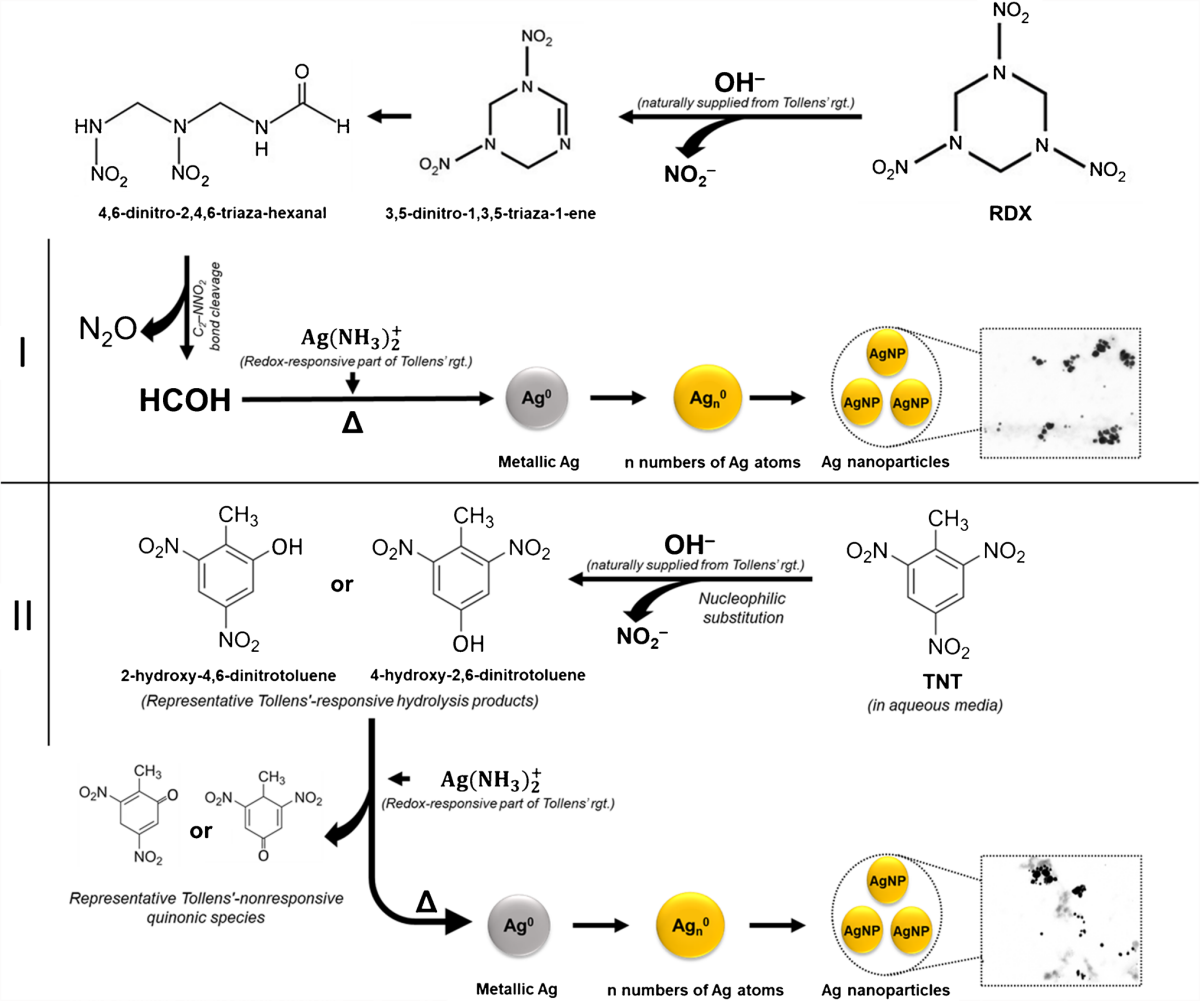
Schematic illustrations of the reaction mechanisms for alkaline hydrolysis of RDX (route I according to Balakrishnan et al*.* [34]) and TNT route II according to Bylaska et al. [40] and Serrano-Lotina et al. [41] in the presence of TR and in situ formation of AgNPs as a result of the reduction of Ag(NH_3_)_2_^+^ by the released HCOH (from RDX) and phenolic degradation products (from TNT)

The possible reaction mechanism for both the in situ degradation of RDX in alkaline solution and the reduction of diamminesilver(I) complex cation to metallic Ag by the formaldehyde released from hydrolytic degradation is as shown in Scheme 1 (route I). Originally, the possible alkaline hydrolysis pathway of RDX in water (pH 10) under the abovementioned experimental conditions was established by the work of Balakrishnan et al. [34] based on the chemical cleavage of the –N−NO_2_ bond of hexyl nitramine functional sites of RDX.

A color test developed by Quesenberry and Lee [26] for the determination of formaldehyde was used to check its formation from RDX degradation and to judge whether it served the proposed sensing mechanism. RDX (and also TNT) were hydrolyzed in the absence and presence of Ag^+^, following the hydrolysis conditions described in the experimental section. The Purpald^®^ assay (active reagent: 4-amino-3-hydrazino-5-mercapto-1,2,4-triazole, 4-AHMT) was applied to RDX and TNT hydrolysates. While no color change was observed with TNT hydrolysates, visible spectra were obtained from RDX hydrolysates, as shown in Figure S7. As seen in Figure S7, in the absence of [Ag(NH_3_)_2_]^+^, absorbance at the characteristic wavelength of 550 nm (of the Purpald chromophore) was obtained as a result of RDX hydrolysis to HCHO, which in turn reacted with 4-AHMT to produce the purple-colored end product with air oxygen. However, in the presence of [Ag(NH_3_)_2_]^+^, formaldehyde released from the alkaline degradation of RDX did not respond to the test, and the peak at a 550 nm wavelength disappeared because HCOH was spent by serving AgNPs formation in accordance with the proposed detection mechanism.

**Fig. 1:**
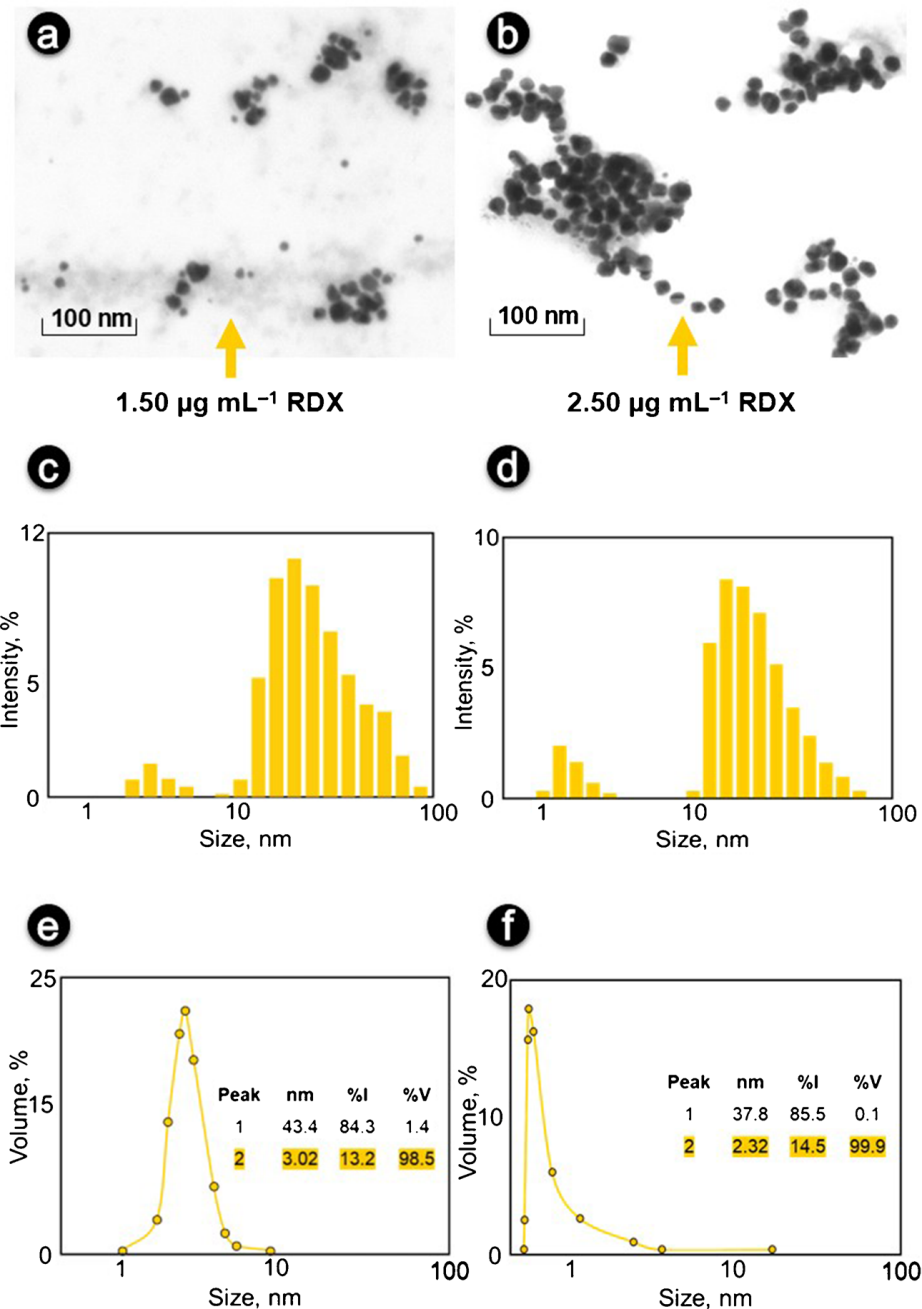
STEM images of in situ formed AgNPs using low (**a)** and high (**b**) conc. of RDX with their DLS-derived intensity (**c**, **d**) and volume (**e**, **f**) distributions (Exp. conditions: [AgNO_3_]_final_ = 2.16 × 10^−4^ mol L^−1^, [NaOH]_final_ = 6.4 × 10^−3^ mol L^−1^, [NH_3_]_final_ = 4.00 × 10^−3^ mol L^−^^1^, incubation temperature and time: 70 °C and 45 min)

The in situ formed AgNPs released from the reduction of [Ag(NH_3_)_2_]^+^ by formaldehyde were characterized by STEM (for imaging) and DLS (for size distribution) techniques, as collectively shown in Fig. 1. Related techniques were used to investigate whether AgNPs formed using both low (1.50 µg mL^−1^) and high (2.50 µg mL^−1^) concentrations of RDX have similar shape and size distribution, regardless of analyte concentration. Accordingly, it was observed that the shape of the AgNPs was spherical at both concentrations of the target analyte, the particles were generally located in close proximity to each other (but not aggregated), and only a larger number of particles were formed in the presence of high analyte concentration than of low concentration, without any remarkable morphological change (Fig. 1a and b). A comparison of the intensity (Fig. 1c and d) and volume/mass distributions (Fig. 1e and f) obtained using low and high concentrations of RDX for DLS analysis is shown in Fig. 1.

Looking at Fig. 1c and d, the two size populations are clearly seen for the two different concentrations: for the low concentration, one at 43.4 nm and the other at 3.2 nm, and for the high concentration, one at 37.8 nm and the other at 2.32 nm. In this case, the larger size group of particles dominates the dispersion for both low and high concentrations, even though they represent only 1.4% (Fig. 1c and e) and 0.1% (Fig. 1d and f) of the entire volume/mass of the sample, respectively. This polydispersity observed in the hydrodynamic size distribution diagram can also be explained by the fact that the AgNPs are located in close proximity to each other (not aggregated), as can be seen from the STEM images, causing unwanted scattering in the DLS device. Thus, when the volume/mass % values used to report the composition data are analyzed, the samples obtained at both low and high concentrations containing AgNPs are observed to be distributed in sizes between 1 and 10 nm with a relative mass composition of 98.5% and 99.9%, respectively. This is in agreement with the results obtained from STEM imaging and supports the fact that approximately 10-nm spherical nanoparticles with uniform distribution were obtained. Thus, AgNPs produced with different concentrations of RDX exhibited similar properties (quasi-spherical, ≤ 10 nm, polydisperse) at a level not creating a significant difference with each other.

The stability of the in situ formed AgNPs was evaluated both by spectrophotometric and zeta potential measurements. Stability experiments of the AgNPs were performed, and absorbance measurements with three repetitions were recorded during 60 min (Figure S8). As seen in Figure S8a, the resulting AgNPs remained stable for 60 min. In addition, no significant decrease or wavelength shift was observed in the absorbance values obtained after measurements for 3 days (Figure S8b). For further evaluation, the ζ-potential of the AgNPs colloidal solution was also measured. As is well known, the larger the value of ζ-potential, especially more than ± 30 mV, the higher the stability of the related nanomaterials will be due to the strong electrostatic repulsion to alleviate their aggregation [42]. Thus, the resulting AgNPs with −35.37 mV ζ-potential are highly stable.

A literature survey to investigate the type of TNT degradation product responding to the system revealed that the common hydrolysis product of RDX and TNT is nitrite [35], which was exploited for the colorimetric determination of nitramine and nitroaromatic compounds [2]. The oldest and best known method for the determination of nitrite is the conventional Griess method. Following the recommended hydrolytic degradation conditions, TNT and RDX were hydrolyzed separately in the presence and absence of [Ag(NH_3_)_2_]^+^. The Griess test (active reagent: a mixture of 0.1% *N*-(1-naphthyl)-ethylenediamine dihydrochloride solution and 1% sulfanilic acid prepared in 5% H_3_PO_4_) was applied to TNT and RDX hydrolysates suitably neutralized with acid [27]. When the visible spectra obtained as shown in Figure S9 are examined, the fact that there is almost no decrement in the absorbance intensities obtained at 540 nm supports that nitrite is formed from both TNT and RDX under the recommended conditions, but does not contribute to the formation of AgNPs according to the proposed mechanism. In this regard, a computational prediction study published by Bylaska et al. [40] shed light on the alkaline degradation pathways of TNT and TNT-related possible mechanism of AgNPs formation, where the authors put forward three different plausible reaction mechanisms first proposed by Salter-Blanc et al*.* [43] for the alkaline hydrolysis of TNT in aqueous and non-aqueous media. Of these three mechanisms, the Meisenheimer complex formation is the most likely alkaline hydrolysis scenario for TNT, but this complex has a short lifetime in aqueous systems due to its low water tolerance [10]. It is obvious that the initial products formed as a result of H-abstraction from the aromatic ring of TNT cannot be reductive, but nucleophilic displacement is the most likely reaction scenario to occur thermodynamically in systems containing predominantly water. The hydration of hydroxyl ions in water is strongly exothermic and exergonic (${\Delta G}_{{\text{hyd}}_{{\text{OH}}^{-}}}=-104.5$ kcal mol^–1^). The dielectric constant of water is 78.30 at room temperature, 63.86 at 70 °C (considering the experimental conditions of the proposed method) [44]. For other solvents with smaller dielectric constants (ethanol, acetone, etc.) the hydration energy of the hydroxyl ion will be less and consequently the reaction energies will decrease. Accordingly, the direct nitro-displacement scenario is notable for the TR, which is largely formed in water. In the study, it is claimed that the hydroxyl ion (OH^–^) attacks the nitro group (–NO_2_) in the ring followed by releasing nitrite, and the ring is hydroxylated from the position where nitrite is released. In the light of this nucleophilic substitution mechanism hypothesis, the CUPRAC method developed by Apak et al. [28] was used for recognizing the alkaline hydrolysis products of TNT, assumed to have a phenolic structure. Following the recommended hydrolysis conditions, TNT and RDX were hydrolyzed separately in the absence and presence of [Ag(NH_3_)_2_]^+^. RDX and TNT hydrolysates were neutralized with acid and the original CUPRAC assay {active ingredient: Cu(II)-neocuproine, Cu(II)-Nc} was performed. When the obtained visible spectra were examined, no color change was observed for RDX, while visible spectra emerged from TNT hydrolysates as shown in Figure S10. In the absence of [Ag(NH_3_)_2_]^+^, a non-negligible absorbance at 450 nm was obtained when the CUPRAC method was applied to the TNT hydrolysate generated by adhering to the alkaline strength of the system and prespecified incubation conditions. In the presence of [Ag(NH_3_)_2_]^+^, a significant decrease in absorbance at 450 nm was observed when the CUPRAC method was applied to the TNT hydrolysate. This was interpreted to mean that the possible phenolic degradation products formed from TNT were significantly consumed in the presence of [Ag(NH_3_)_2_]^+^ by serving AgNPs formation through a redox reaction as per the proposed degradation/AgNPs formation mechanism, and the amount of phenolic species remaining unspent generated a CUPRAC signal, albeit low. It should be noted that the original CUPRAC method (at pH 7) does not respond to nitrite ions at low concentrations (in fact, nitrite requires an acidic medium in order to be oxidized); therefore, nitrite formed under the conditions of the proposed method is unlikely to generate a CUPRAC signal. The CUPRAC and Griess signals (Figure S9 and Figure S10, respectively), obtained by application to alkaline hydrolysates of TNT, support the thermodynamically favorable nucleophilic substitution reaction scenario (i.e., simultaneous formation of phenolic species and nitrite) of alkaline hydrolysis of TNT in aqueous systems, predicted in the computational study of Bylaska et al. [40] with experimental support. In the light of all these theoretical/experimental information, the reaction equation (Eq. (4)) between the end product formed from TNT in aqueous alkaline medium and [Ag(NH_3_)_2_]^+^ complex can be shown as follows:

4 $$\begin{aligned}Ag{\left(NH_3\right)}_2^+\;&+\;\left(O_2N\right)_2-Ar-OH\;+\;H_2O\;\rightleftharpoons\;A{g^0}_{\left(s\right)}\;\\&+\;2NH_3\;+\;{\left(O_2N\right)}_2\;-\;Ar=O\;+\;H_3O^+\end{aligned}$$

Here, (O_2_N)_2_–Ar–OH is used to generally denote the Tollens’-responsive phenolic species 2-, 4- or 6-hydroxy-dinitrotoluene likely to be formed from alkaline hydrolysis of TNT in aqueous media, while (O_2_N)_2_–Ar = O represents the oxidized (quinonic) form of the phenolic species (possible Tollens’-nonresponsive quinonic compounds). Polyphenolics may be formed by similar substitution reactions [45]. The plausible reaction mechanism for both in situ degradation of TNT in the natural alkaline environment of TR and the reduction of the diamminesilver (I) complex to metallic Ag by the nitro-phenolic species, (O_2_N)_2_–Ar–OH, is schematized in Scheme 1 (route II). Moreover, both the negatively charged Meisenheimer anions of TNT and the phenolic degradation product, (O_2_N)_2_–Ar–O^–^, in phenolate form would be strongly attracted to the initially formed AgNPs, which have acquired a positive charge due to the adsorption of the cationic complex, [Ag(NH_3_)_2_]^+^, thereby strongly accelerating silver mirror formation reaction, as encountered in the study of Soukupová et al. [46] who experienced a similar enhanced reaction rate of the negatively charged SDS-adsorbed Ag-nuclei growing in the presence of Ag^+^ cations. Sviatenko et al. [45] also investigated the kinetics and products of TNT hydrolysis and degradation in an alkaline medium and concluded that TNT conversion in such medium consists of five alternatives. Of these possible five routes, only direct substitution of –NO_2_ groups by OH^–^ would be expected to produce CUPRAC-responding phenols, where this CUPRAC response would diminish if the other steps gained effect. For example, in the case when two of the other alternatives involving the formation of Meisenheimer or Janowsky anionic complexes were predominant, these large anions would be attracted by the [Ag(NH_3_)_2_]^+^-adsorbed AgNPs, which catalyze further redox reactions (resulting in ring cleavage) to generate AgNPs, and less phenolics would remain in the medium to produce a negligible CUPRAC response.

In situ formed AgNPs produced by the alkaline degradation products of both low (1.28 µg mL^−1^) and high (3.20 µg mL^−1^) concentrations of TNT were also characterized by STEM (for imaging) and DLS (for size distribution) techniques, as collectively shown in Figure S11. The shape of TNT-derived AgNPs at both concentrations is spherical, although in STEM images (Figures S11a & S11b), the nanoparticles are much closer to each other than in RDX. Looking at Figures S10c & S10d, the three and two size populations are clearly seen for the low and high concentrations, respectively; for the low concentration, one at 21.28 nm and the others at 145.8 and 1.23 nm, and for the high concentration, one at 20.72 nm and the other at 1.63 nm. The size group of particles in the range of 10–100 nm dominates the dispersion for both concentrations of TNT, even though they represent only 0.3% (Figures S10c & S10e) and 0.2% (Figures S11d & S11f) of the entire volume/mass of the sample, respectively. It should be noted that not all phenolic –OH substituents are oxidized to the corresponding quinones in the TR-driven “all-in-tube” system for TNT, and the efficiency of this oxidation depends on the number and position of the phenolic groups of the related degradation products, as well as on the overall conjugation level of the phenolic molecule. The limitations of the method may be the difficulty of oxidation of phenolic species (derived from TNT degradation in aqueous alkaline media) having a standard potential greater than 0.4 V [47] which cannot be oxidized by the TR. For this reason, since the structure of the resulting nitrophenolic degradation products is not fully known, differences in redox potentials, and therefore the possibility of the formation of heterogeneous particles due to the slowness of the reaction, make it possible to see a third peak between 100 and 1000 nm in the hydrodynamic size distribution graph obtained with low concentrations of TNT, unlike that of RDX. However, this resulted in a band broadening of the LSPR maximum of AgNPs obtained with TNT at low concentration (in agreement with the hydrodynamic size distribution plot), but not a wavelength shift of the LSPR maximum of nanoparticles formed with TNT in the appropriate concentration range. Thus, as is apparent from the aforementioned explanations, AgNPs produced with different concentrations of TNT exhibited similar properties (quasi-spherical, ≤ 10 nm, polydisperse) at a level that did not create a significant difference with each other.

### Analytical performance of the TR-driven system

**Fig. 2:**
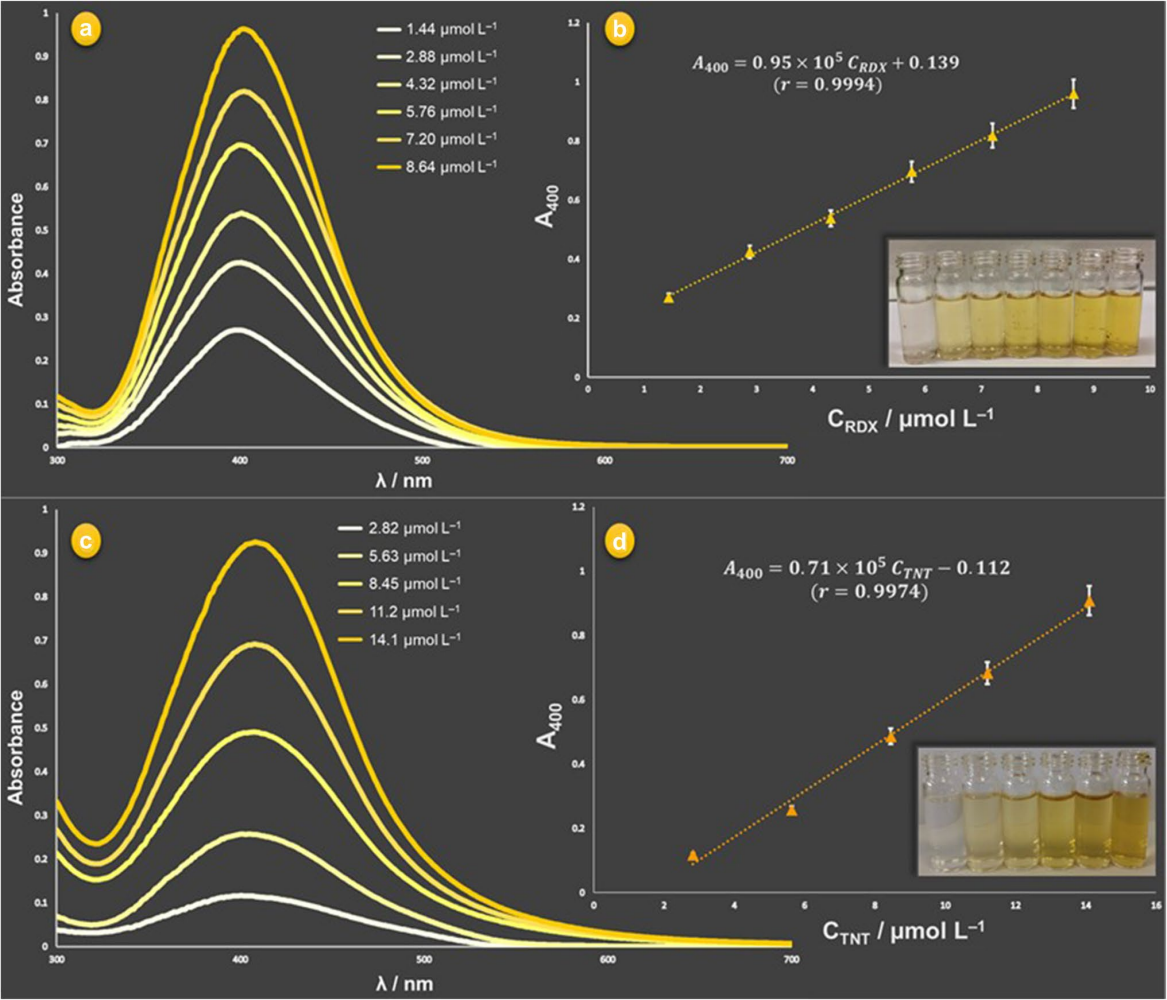
Visible spectra and calibration curves of in situ formed AgNPs applying the proposed procedure to working solutions of RDX (**a**, **b**) and TNT (**c**, **d**) in the final concentration range 1.44–8.64 µmol L^−1^ (0.32–1.92 µg mL^−1^) and 2.82–14.1 µmol L^−1^ (0.64–3.2 µg mL^−1^), respectively, and blank sample solutions with images of the corresponding test tubes (as inset figures) from left to right, containing blank sample solutions (RDX-free and TNT-free) and increasing concentrations of RDX and TNT (experimental conditions: [AgNO_3_]_final_ = 2.16 × 10^−4^ mol L^−1^, [NaOH]_final_ = 6.4 × 10^−3^ mol L^−1^, [NH_3_]_final_ = 4.00 × 10^−3^ mol L^−^^1^, incubation temperature and time: 70 °C and 45 min)

In the proposed work, since AgNPs are produced in an ammonia solution (here TR), the stable [Ag(NH_3_)_2_]^+^ cationic complex adsorbed on AgNPs is responsible for the repulsive effect on NPs, thereby explaining the maximal analytical signal at 400 nm without a wavelength shift for RDX and TNT in a certain concentration range. The absorbance signals of target analyte-induced in situ produced AgNPs are linearly dependent upon concentrations (in µmol L^−1^ range) of the target analytes, RDX and also TNT. As a result, the LSPR absorbance (measured against a blank solution) at 400 nm of AgNPs is proportionally intensified in solutions observed on a color scale scrolling from colorless to yellow. The visible spectra, linear calibration curves (including calibration equations) and color changes (as inset photograph of test tubes) of AgNPs formed with varying concentrations of RDX and TNT for the proposed TR-driven method were collectively shown in Fig. 2.

The obtained absorbance values (remarkably at the same wavelength, suitable for repeatable analytical measurements) were plotted against RDX and TNT concentrations, and the calibration curves were obtained, showing that the change in the absorbance intensity of in situ formed AgNPs had excellent linearity. Analytical performance parameters of the method for RDX and TNT determination are collectively summarized in Table 1. Consequently, the TR-driven system is capable of analyte detection with good sensitivity and precision.

**Table 1:** Analytical performance parameters of the TR-driven sensing system for RDX and TNT

Analyte	Tollens’-responsive degradation product	Slope^*a*^-intercept values of the calibration lines	Linear range^*b*^	LOD^*c*^	LOQ^*d*^	RSD,%^*e*^	
						intra-assay	inter-assay
RDX	formaldehyde	0.95 × 10^5^ + 0.139	1.44–8.64	50.3	168.0	0.43	0.87
TNT	2-, 4-, or 6-OH-DNT	0.71 × 10^5^ − 0.112	2.82–14.1	67.2	224.0	1.38	1.50

^*a*^In mg^−1^ L units

^*b*^In µmol L^−1^ units (final conc.)

^*c,d*^Units at final concentration*.* limit of detection (LOD = 3 *σ*_bl_/*m*, LOQ = 3.3 LOD, *σ*_bl_ denoting the standard deviation of a blank and *m* showing the slope of the calibration line), in mg L^−1^ units (final conc.)

^*e*^Relative standard deviation, as a percentage (*N* = 5)

The robustness of the proposed system can be investigated by deliberately making small experimental changes in varying physical and environmental conditions such as operating wavelength (± 1 nm), temperature (± 1 °C), volume or concentration of reagents. For this purpose, the developed TR-driven system was applied to RDX and TNT solutions of the same concentration with three replicates in two different laboratories, and the visible region spectra of the solutions were measured with two different UV/vis spectrophotometers. As can be seen in Figure S12, such a change in the experimental procedure had no significant effect on the absorbance values observed for RDX and TNT. The relative standard deviation values were found to be < 3%, indicating that the developed system is robust.

### Extraction-based recovery of RDX from TNT-contaminated soil samples

When it comes to the use of nitro-explosives for both military purposes and terrorist activities (e.g*.*, the use of PETN to enhance the explosive effect along with TNT and RDX, possibly Composite B, in the 2016 Beşiktaş-Istanbul terror attacks), RDX and TNT are the two energetic substances most likely to be found together. Therefore, it is important to determine RDX and TNT both separately and simultaneously with the proposed method. Thus, inspired by the work of Kang et al. [48], extraction-based separation of RDX was proposed for the determination of RDX and/or TNT in RDX/TNT-contaminated soil samples or debris collected from crime scenes. For this purpose, organic solvents displaying a limited solubility of RDX, whereas a relatively high solubility of TNT, were sought. Experiments were conducted with soil samples synthetically contaminated with RDX/TNT using ethanol and toluene; the most efficient separation was achieved with toluene. TNT can dissolve in solvent structures similar to itself, such as toluene (67 g/100 mL, 25 °C) [49], while RDX belongs to the naphthenic compounds class, having a completely symmetric structure with almost insolubility in toluene (0.02 g/100 mL, 25 °C) [50]. Therefore, toluene was selected for organic solvent extraction of TNT at room temperature. Since toluene forms a phase with water, the compatibility of toluene-extracted TNT with a highly aqueous reaction medium was achieved with ethanol. The findings for the recovery of RDX and TNT from contaminated soil samples are summarized in Table S1. Accordingly, RDX was recovered from RDX and TNT-contaminated soil with a yield higher than 90%.

### Evaluation of selectivity and possible interference effects

**Fig. 3:**
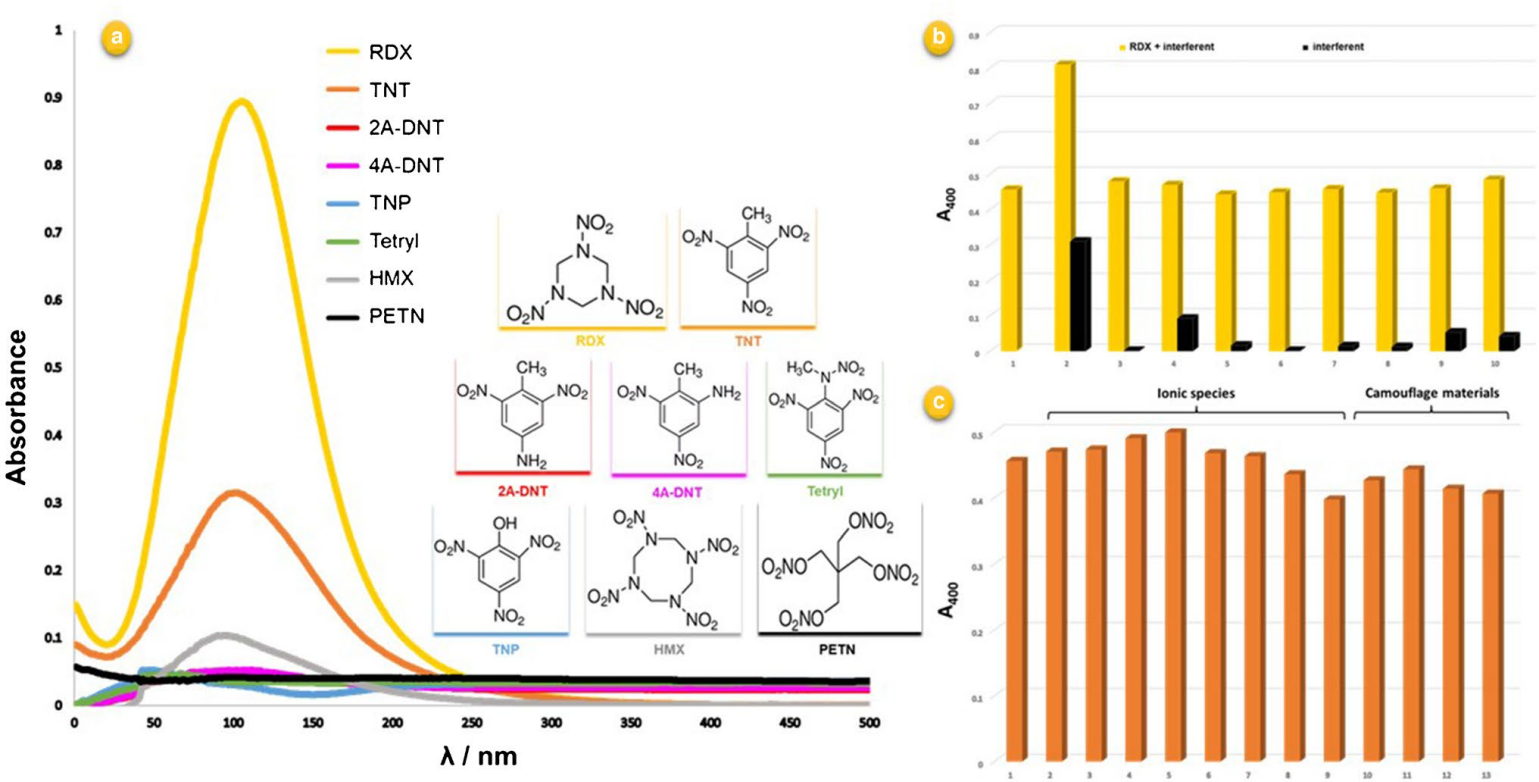
**a** Comparison of the visible spectra obtained from the proposed TR-driven system in the presence of RDX, TNT, and some possible interfering energetic compounds, whose chemical structures are shown on the right side; **b** absorbance intensity of the TR-driven system in the presence of RDX (yellow bars) and in the absence of RDX (black bars) with different types of energetic materials (ten-fold): RDX (only) (1), TNT (2), TNT after toluene extraction (3), HMX (4), TNP (5), PETN (6), 2A-DNT (7), 4A-DNT (8), tetryl (9), and humic acid (in soil samples) (10); **c **absorbance intensity of the TR-driven system in the presence of RDX with possible interferent ionic species and camouflage materials (ten-fold): RDX (only) (1), Ca^2+^ (2), Mg^2+^ (3), Cu^II^ (4), Fe^II^ (5), Al^III^ (6), SO_4_^2−^ (7), Cl^−^ (8), CO_3_^2−^ (9), acetylsalicylic acid (aspirin) (10), D-(+)-glucose (for one-fold of RDX) (11), aspartame (12), household detergent (for one-fold of RDX) (13)

The selectivity of the TR-driven system toward the target analyte RDX and TNT was investigated for other alkaline-degradable energetic compounds, mainly 2 A-DNT, 4 A-DNT, tetryl, TNP, HMX, and PETN, for the soil component humic acid (in soil samples), and also for some ionic species and camouflage materials (Fig. 3).

The visible spectra of the system obtained with the aforementioned energetics individually were compared with that of RDX. As seen in Fig. 3a, it was observed that in situ formation of AgNPs only arose from the presence of RDX and TNT, and there was no remarkable change in absorption intensity by the other energetic compounds. Among nitro-explosives, it is also important to examine the interference effect of the nitramine compound HMX, the structural analog of RDX. In the literature, formaldehyde is reported among the degradation products of HMX under alkaline hydrolysis conditions. However, the degradation of HMX is considerably slower than that of RDX, and the ambient pH required for keeping its degradation rate and efficiency at a reasonable level is 12.3, while for RDX, this edge pH is 10, which is regulated to be approximately 10–11 using a reasonable amount of NaOH. Therefore, it is not possible for HMX to respond to the system unless pH is brought to 12 and above; on the other hand, at this pH (pH ≥ 12), RDX is likely to degrade rapidly and uncontrollably, and the Tollens’-responsive degradation product formaldehyde is converted to Tollens’-nonresponsive end products such as formate/ammonia by special reactions such as aldol condensation/Cannizzaro reaction, resulting in a sudden drop in the analytical signal, as shown in Figure S2 [34]. In addition, the interference effect of energetics such as 2 A-DNT, 4 A-DNT, tetryl, TNP, and PETN, both alone and in admixture with RDX, were investigated and RDX recovery values were calculated. According to the data shown in Table S2, the recovery values of RDX in the presence of all relevant energetic substances were in the range of 96.9 ± 0.9% to 106.1 ± 3.2%. The bar diagram for the interference effect results for the recovery of the target analyte RDX along with TNT and other energetic substances is shown in Fig. 3b. The U.S. Cold Regions Research & Engineering Laboratory (CRREL) method is interfered with by the presence of soil humic acids requiring background correction [2], as compared to the developed method unaffected by humates (as these substances are multivalently anionic at high pH, and therefore not extracted into acetone). Thus, soil humates did not yield any interference to the proposed method of RDX assay with ≥ 90% recovery values. The feasibility of the proposed method was also examined in the presence of common ionic species (metal cations and anions) and camouflage materials that can be handled by passengers as personal items in hand-carried luggage. The bar diagram of the interference effects for target analyte RDX along with ionic species and camouflage materials is shown in Fig. 3c. The recovery values given in Table S3 for ionic species and camouflage materials were in the range of 91.2 ± 0.8–103.6 ± 0.9% and 93.4 ± 2.5–108.4 ± 4.9%, respectively.

### Analysis of some synthetic/real munition formulations

Absorbance values obtained for synthetically prepared binary mixtures containing varying mass ratios of RDX and TNT (Table S4) were compared with those obtained individually from the two explosives. Data in Table S4 show that the proposed system yields results for RDX and TNT in accordance with the principle of additivity of absorbances, indicating the lack of chemical deviations from Beer’s law. The developed method was also applied to two different synthetically prepared TNT-based munition mixtures, amatol (TNT + NH_4_NO_3_) and pentolite (TNT + PETN), and as shown in Table S5, recovery values close to 100% were achieved. Composition B (Comp B) is the most well-known formulation combining RDX and TNT. Therefore, for the purpose of real sample analysis, the linear calibration equation (${\text{A}}_{400}=0.296 {\text{C}}_{\text{CompB}}+0.205, r=0.9961$) pertaining to the visible spectra of the resulting AgNPs was constructed for increasing Comp B concentrations with the application of the proposed method to varying volumes of 32.0 µg mL^−1^ Comp B solution by first utilizing the total analyte signal of RDX and TNT (Figure S13). The results show that for the real sample Comp B, the system responds additively to RDX and TNT and can be reliably used for Comp B determination. In addition, Comp A5 solution was determined using normal calibration method, yielding 91.86% recovery for RDX. Finally, Octol was analyzed by the method of standard additions, and calibration plots and line equations were generated for TNT standards ($A_{400}\;=\;2.88\;\times\;10^{-1}\;C_{TNT/\mu g\;mL^{-1}}\;-\;0.082,\;r\;=0.9904$) and 32.0 µg mL^−1^ TNT-spiked Octol samples ($A_{400}\;=\;2.49\;\times\;10^{-1}\;C_{\left(Octol\;+\;TNT\right)/\mu g\;mL^{-1}}\;-\;0.270,\;r\;=0.9944$) (Figure S14). Accordingly, the initial TNT amount for Octol was calculated as 32.25 µg mL^−1^. As the calibration curves are parallel to each other, there is no interference effect by HMX or other impurities present in the real composition of Octol.

### Statistical comparison of the recommended method against a reference LC − MS/MS technique

The data obtained from the developed method and the reference LC–MS/MS method [29] using five different RDX standard solutions at the same concentration, as well as RDX solutions obtained by acetone extraction from clean sandy soil samples contaminated with three different RDX solutions at the same concentration, were subjected to *t-*and *F-*tests in terms of accuracy and precision in Table S6. Comparison with both RDX standard solutions and RDX-contaminated soil samples showed no significant difference at the 95% confidence level for *t-* and *F-*tests, respectively.

### Comparison of the developed method with other colorimetric methods for RDX detection

A comparison of the proposed method with other reported colorimetric methods for RDX detection, including the possible interference effect of TNT, is summarized in Table S7. In contrast to the existing systems in the literature, an AgNPs formation-based mechanism with LODs at the nmol L^−1^ level using the traditional Tollens’ reagent was implemented to detect explosives for the first time. In addition, the assay is superior in that it does not require pre-hydrolysis for RDX, allowing direct determination in practice, and offers an alternative to the removal/detection studies developed for TNT based on the Meisenheimer/Janowsky anions formation mechanism with low water tolerance. The assay also offers a more efficient and high-throughput alternative for RDX, as opposed to the famous Griess assay of nitrite determination, which is subject to interference from nitrates and other nitro-compounds.

## Conclusion

Apart from the predominantly used aggregation and enzyme-like catalytic activity-induced nanoparticle-based approaches, the lack of any approach based on nanoparticle formation for colorimetric sensing of explosives led to the development of a colorimetric assay with an unusual working principle for RDX and also TNT. This work involves a revival of the Tollens’ method, which has been used for centuries for the detection of aldehydes with the silver mirror reaction performed in an Ag(I)–NH_3_ system containing NaOH, for producing controlled-size AgNPs from RDX under alkaline conditions. In line with this motivation and target, the main component of the traditional TR, which naturally contains OH^–^, is the diamminesilver (I) cationic complex {Ag(NH_3_)_2_^+^/Ag^0^ = 0.38 V} with a reduction potential half that of the silver(I) ion (Ag^+^/Ag^0^ = 0.799), allowing direct determination of RDX without responding to HMX, as the decomposition product (formaldehyde) released by alkaline hydrolysis of RDX with OH^–^ in the medium kinetically induces faster in situ AgNPs formation than HMX. Since our system also enabled TNT determination (previously estimated with the formation of water-intolerant Meisenheimer complexes of varying colors), both RDX and TNT could be assayed by AgNPs formation, though with different mechanisms. Accordingly, for the possible degradation products of alkaline-hydrolyzed RDX and TNT that may serve the AgNPs formation mechanism, three simple colorimetric tests; namely, the Purpald® test was used for the detection of formaldehyde, the Griess test for the detection of nitrite, and the CUPRAC test for the detection of phenolic species. Thus, the assertion that formaldehyde from RDX and phenolic species from TNT cause in situ AgNPs formation was strengthened using simple color tests. The STEM imaging technique was used under the proposed experimental conditions to distributionally characterize the in situ formed AgNPs. It was observed that in the presence of low and high concentrations of RDX and TNT, nanoparticles with a spherical shape and a high proportion of sizes between 1 and 10 nm and uniform distribution were obtained. The LSPR absorbances (at the same stable wavelength) of AgNPs derived from RDX and TNT were additive, enabling the analysis of synthetic and real mixtures, but the two explosives could be individually determined by preliminary extraction of TNT with toluene using the proposed TR-driven “all-in-a-tube” system. Simultaneous hydrolysis and direct detection of RDX were performed in the same TR-driven system, without a need for pre-hydrolysis/neutralization processes. This simultaneous determination capability of the two most abundant nitro-explosives with a simple NP formation-based approach is believed to bring a new breath to the identification/quantification of energetic materials by guiding criminological and environmental investigations.

## Supplementary Information

Below is the link to the electronic supplementary material.

The Supplementary Information is available free of charge and the file contains: Abbreviations; Preparation of solutions; Procedures of Purpald, Griess, and CUPRAC colorimetric assays used to elucidate the detection mechanism of the system, LC − MS/MS conditions for RDX determination; Supplementary Figures, Supplementary Schemes, Supplementary Tables, and Supplementary References.

ESM 1 (1210 KB DOCX)

## Data Availability

No datasets were generated or analysed during the current study.
