# Supplementary material for: “All-in-a-tube” detection of RDX and TNT: old silver mirror reaction revived for nitro-explosive quantification
Source: Mikrochim Acta. 2025 May 8;192(6):344. doi: 10.1007/s00604-025-07195-w (PMC12062072; doi:10.1007/s00604-025-07195-w)
Supplement: Supplementary file 1 — (1210 KB DOCX) [file 604_2025_7195_MOESM1_ESM.docx]

**ELECTRONIC SUPPLEMENTARY INFORMATION**

**“All-in-a-tube” detection of RDX and TNT: old silver mirror reaction revived for nitro-explosive quantification**

Selen Durmazel,^a^ Ayşem Üzer,^a,*^ Reşat Apak^a,b,^*

^a^Analytical Chemistry Division, Department of Chemistry, Faculty of Engineering, Istanbul University-Cerrahpaşa, Avcilar 34320, Istanbul, Türkiye

^b^Turkish Academy of Sciences (TUBA), Çankaya 06670, Ankara, Türkiye

*Corresponding author: Ayşem Üzer, E-mail: [auzer@iuc.edu.tr](about:blank)

*Co-corresponding author: Reşat Apak, E-mail: [rapak@istanbul.edu.tr](about:blank); Tel.: +90-212-4737028

Table of Contents

[Abbreviations S2](#_Toc196140217)

[Materials and Chemicals S3](#_Toc196140218)

[Instrumentation S3](#_Toc196140219)

[Preparation of Solutions S4](#_Toc196140220)

[Colorimetric assays used to elucidate the detection mechanism of the system S5](#_Toc196140221)

[Extraction-based recovery process of RDX and TNT S6](#_Toc196140222)

[Reference LC−MS/MS Conditions for RDX Determination S7](#_Toc196140223)

[Supplementary Schemes S8](#_Toc196140224)

[Supplementary Figures S10](#_Toc196140225)

[Supplementary Tables S24](#_Toc196140226)

[Supplementary References S31](#_Toc196140227)

# Abbreviations

1,3,5-trinitroperhydro-1,3,5-triazine (RDX), 2,4,6-trinitrotoluene (TNT), 3-nitro-1,2,4-triazole-5-one (NTO), silver nanoparticles (AgNPs), liquid chromatography–tandem mass spectrometry (LC–MS/MS), 4-amino-3-hydrazino-5-mercapto-1,2,4-triazole (4-AHMT), Cupric reducing antioxidant capacity (CUPRAC), 2,9-Dimethyl-1,10-phenanthroline (Neocuproine, Nc), sodium hydroxide (NaOH), aqueous ammonia (aq. NH_3_), 2-amino-4,6-dinitrotoluene (2A-DNT), 4-amino-2,6-dinitrotoluene (4A-DNT), 2,4,6-trinitrophenylmethylnitramine (Tetryl), 1,3,5,7-tetranitro-1,3,5,7-tetraazacyclooctane (HMX), 2,4,6-trinitrophenol (TNP), pentaerythritol tetranitrate (PETN), ammonium nitrate (NH_4_NO_3_, AN), sodium carbonate (Na_2_CO_3_), gold nanoparticles (AuNPs), nitrous acid (HNO_2_), 3,3',5,5',-tetramethylbenzidine (TMB), hydrochloric acid (HCl).

# Materials and Chemicals

The energetic materials used throughout the study, RDX (containing 85% active matter), TNT (pure), 2,4,6-trinitrophenylmethylnitramine (Tetryl, pure), 2,4,6-trinitrophenol (TNP, pure), 2-amino-4,6-dinitrotoluene (2A-DNT, pure), 4-amino-2,6-dinitrotoluene (4A-DNT, pure), 1,3,5,7-tetranitro-1,3,5,7-tetraazacyclooctane (HMX, pure), pentaerythritol tetranitrate (PETN, pure), Composite B (containing 60% RDX, 39% TNT, and 1% wax), Octol (containing 70% HMX and 30% TNT) and Composite A5 (containing 99% RDX and 1% filler material) were kindly provided in low amounts by the Mechanical and Chemical Industry Corporation of Türkiye (Makine Kimya Endüstrisi Kurumu-MKEK; Ankara, Türkiye) from previous projects.

Silver nitrate (AgNO_3_) and humic acid sodium salt were obtained from Sigma-Aldrich (Steinheim, Germany). Aqueous ammonia solution (NH_3_, 25% w/w) and sodium hydroxide (NaOH) were purchased from Merck (Darmstadt, Germany). Acetone was obtained from Riedel-de Haën - Honeywell Research Chemicals (Charlotte, South Carolina, USA). Ethanol (EtOH) was ordered from ISOLAB (Eschau, Germany). Glass fiber/poly(ethylene terephthalate) microfilter (CHROMAFIL GF/PET-45/25) was purchased from MachereyNagel (Düren, Germany). As certified reference materials for real sample analysis, clean sandy soil (Non-Polluted Blank Soil #1, CLN SOIL-1) was purchased from RTC (Laramie, WY, USA).

# Instrumentation

The obtained visible spectra and absorption measurements were recorded using a Shimadzu UV1800 and UV1900i ultraviolet−visible spectrophotometers (Kyoto, Japan) monitored in the range of 300–800 nm. Incubation of the RDX- and TNT-containing standard and/or samples was performed using the Wisd WiseBath water bath (Wertheim, Germany). Scanning transmission electron microscopy (STEM) measurements were carried out by using a FEI Quanta FEG 450 (Hillsboro, Oregon, USA). Dynamic light scattering (DLS) measurements were performed using a 90Plus Particle Size Analyzer (Brookhaven Instrument, USA) equipped with a 35 mW HeNe laser at a temperature of 25.0 ± 0.2 °C in water. The developed method was statistically compared and validated against a literature liquid chromatography−tandem mass spectrometry (LC−MS/MS) method using a Shimadzu-8040 liquid chromatograph (Kyoto, Japan) equipped with two mass spectrometers containing an electrospray ionization source with a quadrupole mass analyzer. LC was equipped with a Restek Ultra AQ C18 (3 mm by 100 × 2.1 mm inner diameter) column.

# Preparation of Solutions

For the preparation of the components of the Tollens’ reagent; 1.0×10^–2^ mol L^–1^ AgNO_3_ stock solution was prepared daily in water. 0.10 mol L^–1^ of NaOH stock solution was prepared in ultrapure water. AgNO_3_ and NaOH solutions used in optimization studies within the concentration range of 1.0 – 2.0×10^–3^ mol L^–1^ and 0,01 – 0,09 mol L^–1^, respectively, were prepared by taking appropriate volumes from the respective stocks and diluting with water. 2.0×10^–2^ mol L^–1^ concentration of aq. NH_3_ solution was prepared in water and used directly. Stock solutions of RDX and TNT containing 2000 mg L^–1^ active matter was prepared in acetone. 32.0 µg mL^–1^ of working solutions for RDX and TNT were prepared by taking appropriate volumes of corresponding stock solutions and diluting with water. Stock and diluted solutions of Octol (70% HMX + 30% TNT), C4 (99% RDX + %1 inert filler), and Composite B (60% RDX + 39% TNT + 1% wax) as the real military-purpose explosive mixtures were also prepared in acetone to 2000 µg mL^–1^. A series of synthetic mixtures containing varying mass ratios of RDX and TNT (1:1, 1:2, 1:3, 2:1, 2:3, 3:2 and 3:1, RDX:TNT, w/w) in solution phase were prepared using suitable mixing of 2000 µg mL^–1^ of these corresponding stock solutions. Solution forms of TNT – based munition formulations (containing 32.0 mg L^–1^ TNT); Amatol (80% NH_4_NO_3_ + 20% TNT) and Pentolite (50% PETN + 50% TNT) were artificially prepared in ethanol. The proposed assay was applied to all of these formulations and recovery values of RDX and/or TNT were calculated. Stock solutions of all energetic substances used throughout the study, prepared in an appropriate organic solvent, were stored at –18 ^o^C for further experiments. The working solutions of RDX for calibration curves of LC–MS/MS analyses were prepared daily within the concentration range of 25.0 – 400.0 µg L^–1^ by diluting with acetonitrile from the corresponding stock solution at 2000.0 mg L^–1^ in acetone.

# Colorimetric assays used to elucidate the detection mechanism of the system

***Purpald^®^ assay.*** Adhering to the alkaline conditions of the recommended procedure {both in the presence and absence of [Ag(NH_3_)_2_]^+^}, 1.0 mL of high and low conc. RDX or TNT sample solutions incubated/hydrolyzed (under the certain conditions of the recommended procedure) in the conditions of the recommended procedure is taken into a test tube and 1.0 mL of 34.0 mmol L^–1^ Purpald^®^ reagent (in 2.0 mol L^–1^ NaOH) is added. The test tubes are kept at room temperature for 20 min, followed by addition of 1.0 mL 33.0 mmol L^–1^ NaIO_4_ (4-AHMT, in 0.20 mol L^–1^ NaOH). Absorbance values were measured at 550 nm against a blank solution (excluding hydrolyzed RDX and TNT) using a UV/vis spectrophotometer.^1^

***Griess assay.*** Adhering to the alkaline conditions of the recommended procedure {both in the presence and absence of [Ag(NH_3_)_2_]^+^}, 1.0 mL of high and low conc. RDX or TNT sample solutions incubated/hydrolyzed (under the certain conditions of the recommended procedure) and neutralized (using 0.1 mol L^–1^ H_2_SO_4_) is taken into a test tube and 3.0 mL of Griess reagent consisting of 1.0% sulfanilamide + 0.1% *N*-1-naphthylethylenediamine dihydrochloride (in 5.0% H_3_PO_4_) is added. Absorbance values were measured at 540 nm against a blank solution (excluding hydrolyzed RDX or TNT) using a UV/vis spectrophotometer.^2^

***CUPRAC assay.*** Adhering to the alkaline conditions of the recommended procedure {both in the presence and absence of [Ag(NH_3_)_2_]^+^}, 1.0 mL of high and low conc. RDX or TNT sample solutions incubated/hydrolyzed (under the certain conditions of the recommended procedure) and neutralized (using 0.1 mol L^–1^ H_2_SO_4_) was used for applying the main CUPRAC assay. For this respect, 1.0 mL 1.0×10^–2^ mol L^–1^ Cu(II) soln., 1.0 mL 7.5×10^–3^ mol L^–1^ Nc ethanolic soln., 1.0 mL 1.0 mol L^–1^ ammonium acetate (pH ~ 7.0), 1.0 mL sample solution, and 0.1 mL ultrapure water were taken into a test tube, respectively. After 30 min. incubation at room temperature, absorbance values were measured at 450 nm against a blank solution (excluding hydrolyzed RDX or TNT) using a UV/vis spectrophotometer.^3^

# Extraction-based recovery process of RDX and TNT

In order to determine RDX and TNT separately, each contaminated soil sample was first treated with 1.0 mL of toluene and mixed with a vortex for 1 minute. It was then centrifuged at 5000 rpm for 5 minutes and the supernatants were filtered using a CHROMAFIL GF/PET-45/25 filter. Four milliliters of ethanol were added to the toluene extracts containing TNT and the final volume was completed to 25.0 mL with ultrapure water. Soil samples containing undissolved RDX crystals were treated with 2.5 mL acetone:ethanol (1:4, v/v) mixture, mixed with a vortex for 1 min, then centrifuged at 5000 rpm for 5 min, and the supernatants were filtered using a CHROMAFIL GF/PET-45/25 filter. Analysis of the soil samples was carried out by applying the recommended assay to the solutions.

# Reference LC−MS/MS Conditions for RDX Determination

The literature LC−MS/MS method^4^ was applied to RDX detection with some modifications, and the analyses of RDX samples were done using the apparatus described in a previous study of our research group.^5^ An LC–MS/MS apparatus equipped with a Restek Ultra AQ C18 (3 mm × 100 mm × 2.1 mm ID) reversed phase column was used. At a flow rate of 0.30 mL min^–1^, the column was eluted with the isocratic elution hold at the mobile phase composition of 5% mobile phase A of 5.0 mmol L^–1^ ammonium acetate in water and 95% mobile phase B of 5.0 mmol L^–1^ ammonium acetate in methanol for 3 min. The injection volume and the column oven temperature were 15.0 µL and 40.0 °C, respectively. LC−MS/MS in multiple reaction monitoring (MRM) mode was carried out exploiting the negative electrospray ionization, and the ionization voltage was 3.5 kV. The product ion and precursor ion were 281.1 m/z and 46.15 m/z for RDX, respectively (collision energy: 10.0 V).^4^

# Supplementary Schemes


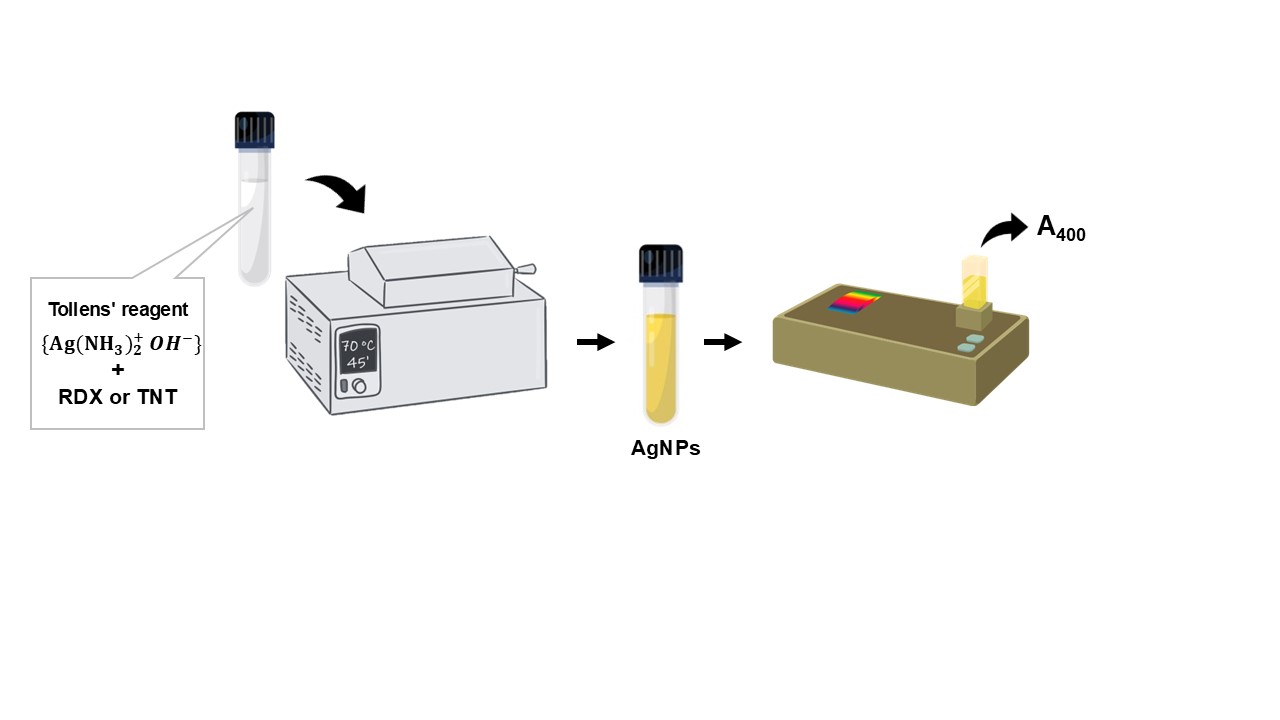


**Scheme S1.** Schematic illustration of applying the recommended "all-in-a-tube" procedure for RDX or TNT determination.


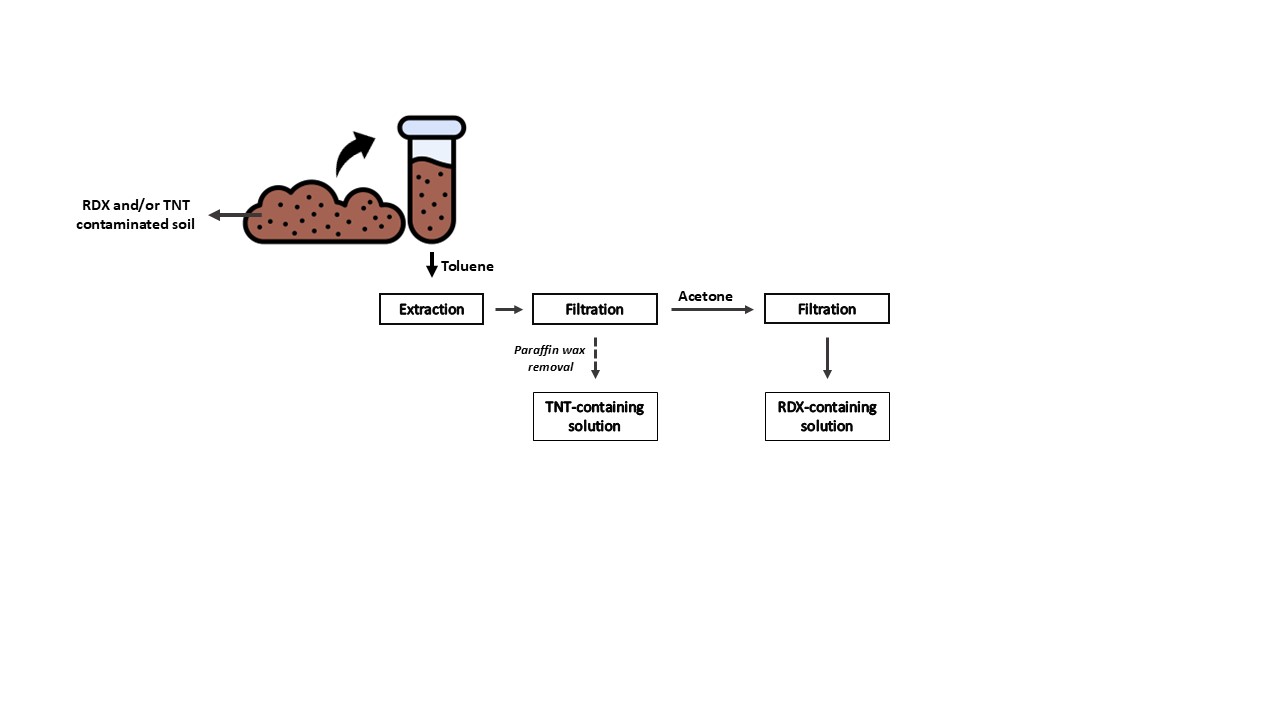


**Scheme S2.** Flow diagram of TNT removal from contaminated soil and recovery steps of RDX.

# Supplementary Figures


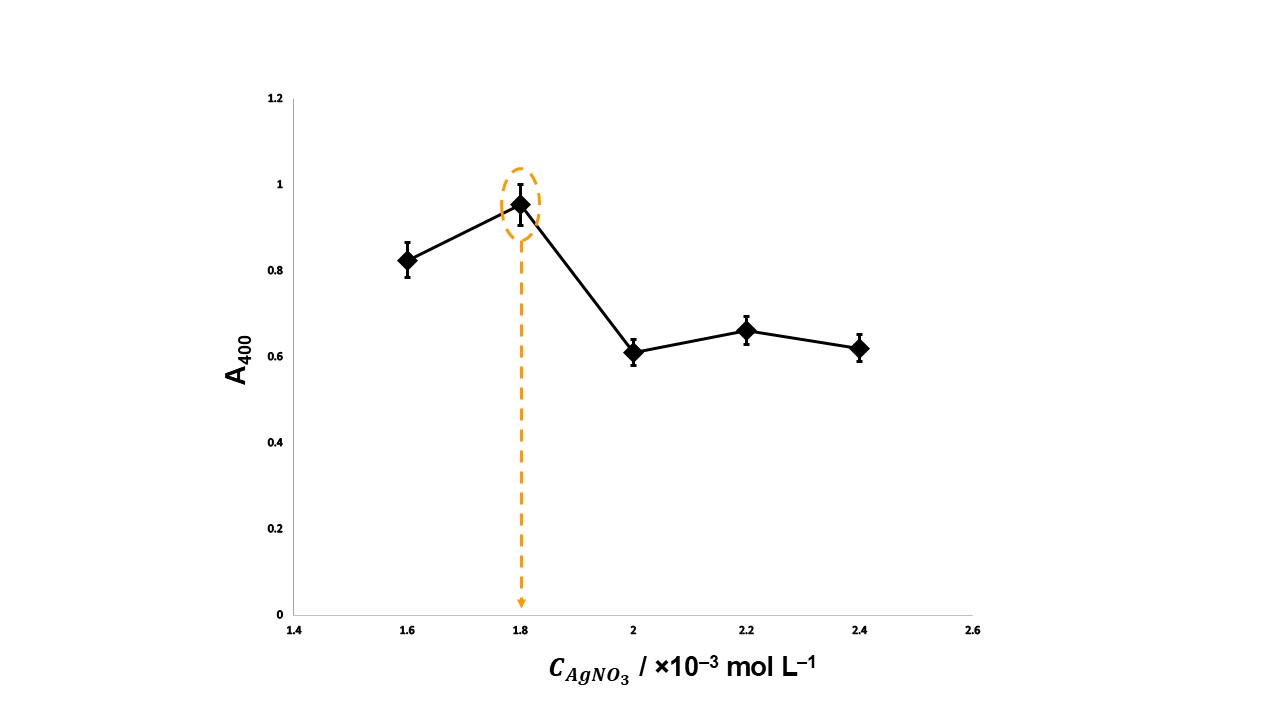


**Figure S1.** Optimization graph of absorbance values obtained by applying the method with Ag-diamine complex prepared using silver nitrate solution at varying concentrations (1.60 – 2.40 mmol L^–1^) {Exp. conditions: [NaOH]_final_ = 8.0×10^–3^ mol L^–1^, [NH_3_]_final_ = 4.00×10^–3^ mol L^–1^, [RDX]_final_ = 10.0 µg mL^–1^, incubation temperature and time: 60 ^o^C and 20-min}.


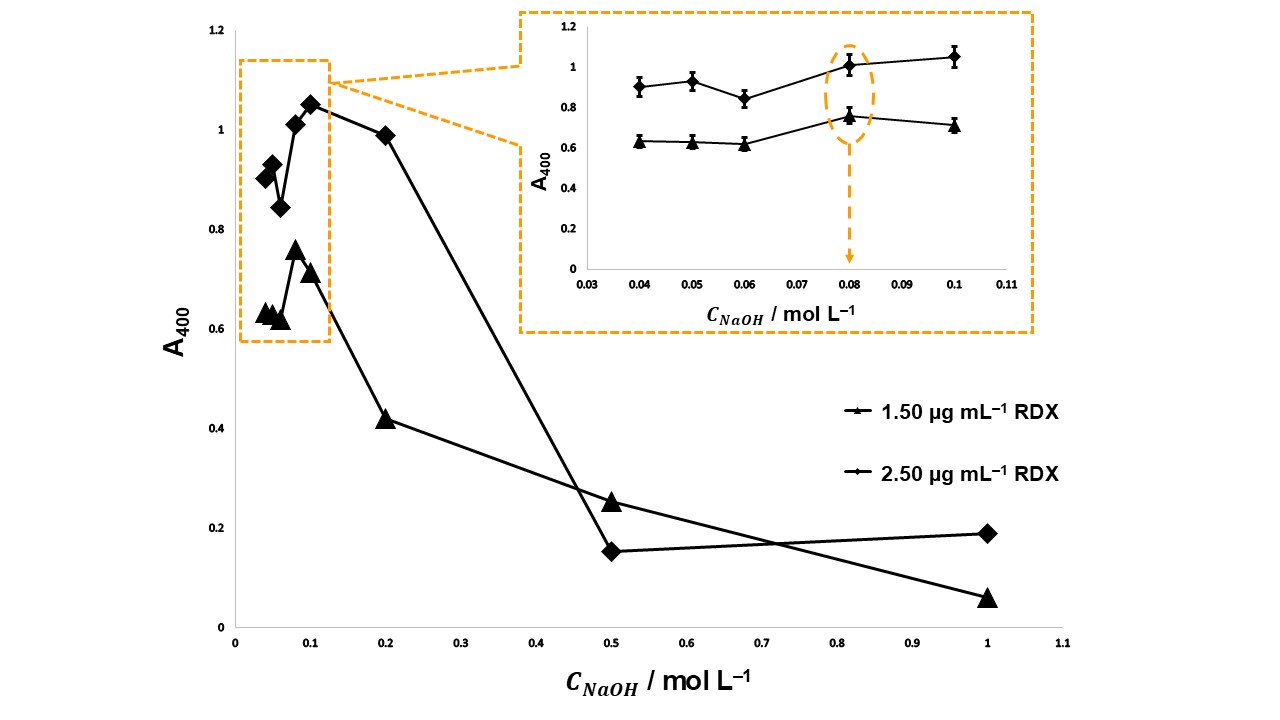


**Figure S2.** Optimization graph of absorbance values obtained by applying the method to 1.50 and 2.50 µg mL^-1^ RDX solutions with Ag(I)-diamine complex prepared using varying concentrations of NaOH solution (0.01 – 0.20 mol L^–1^, initial conc.) {Exp. conditions: [AgNO_3_]_final_ = 2.16×10^–4^ mol L^–1^, [NH_3_]_final_ = 4.00×10^–3^ mol L^–1^, [RDX]_final_ = 10.0 µg mL^–1^, incubation temperature and time: 60 ^o^C and 20-min}.


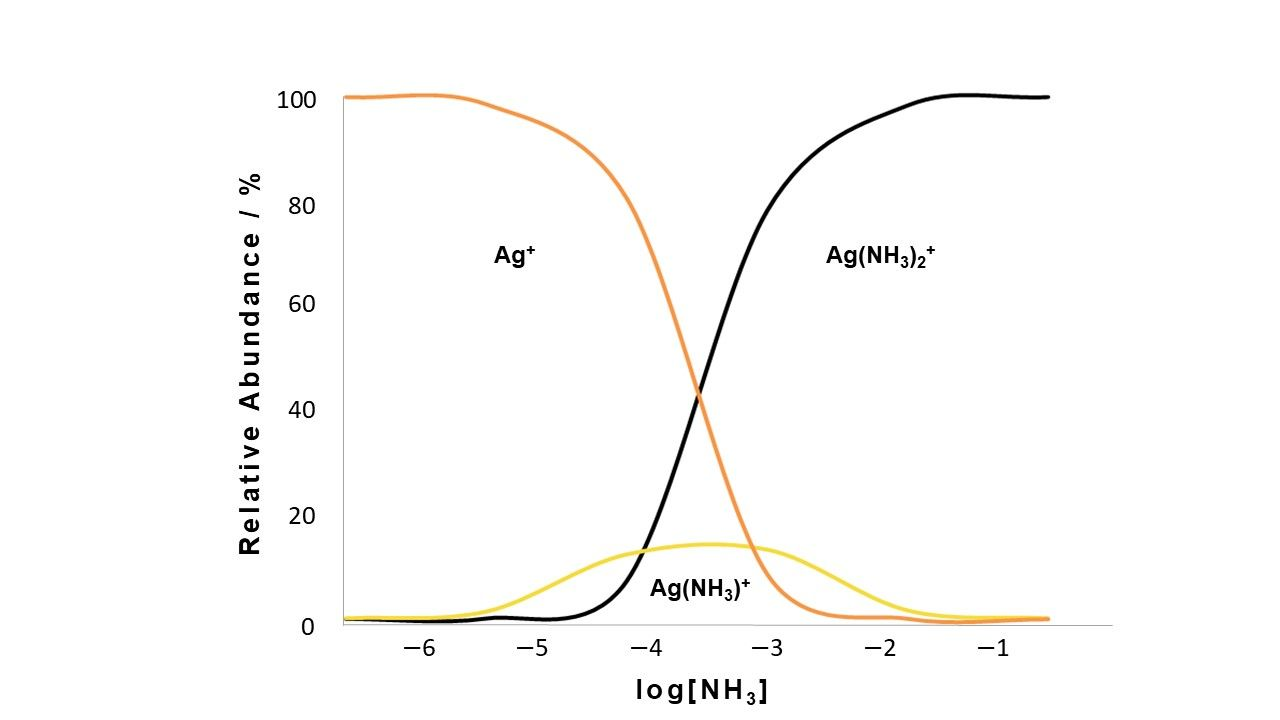


**Figure S3.** The species distribution curve for the formation of Ag(I)–ammine complexes.


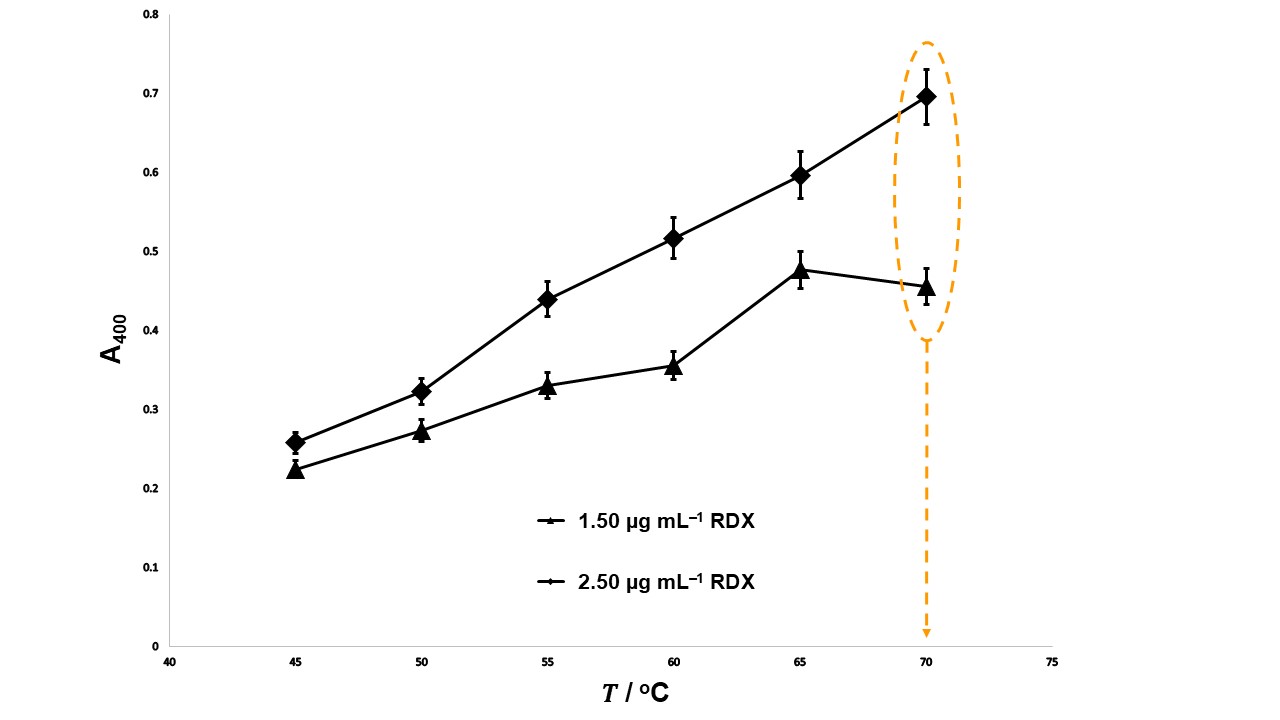


**Figure S4.** Optimization graph of absorbance values obtained at different temperatures (45.0 – 70.0 ^o^C) by applying Ag(I)-diamine complex to 1.50 and 2.50 µg mL^–1^ (final concent.) RDX solutions {Exp. conditions: [AgNO_3_]_final_ = 2.16×10^–4^ mol L^–1^, [NaOH]_final_ = 6.4×10^–3^ mol L^–1^, [NH_3_]_final_ = 4.00×10^–3^ mol L^–1^, [RDX]_final_ = 10.0 µg mL^–1^, incubation time: 20-min}.


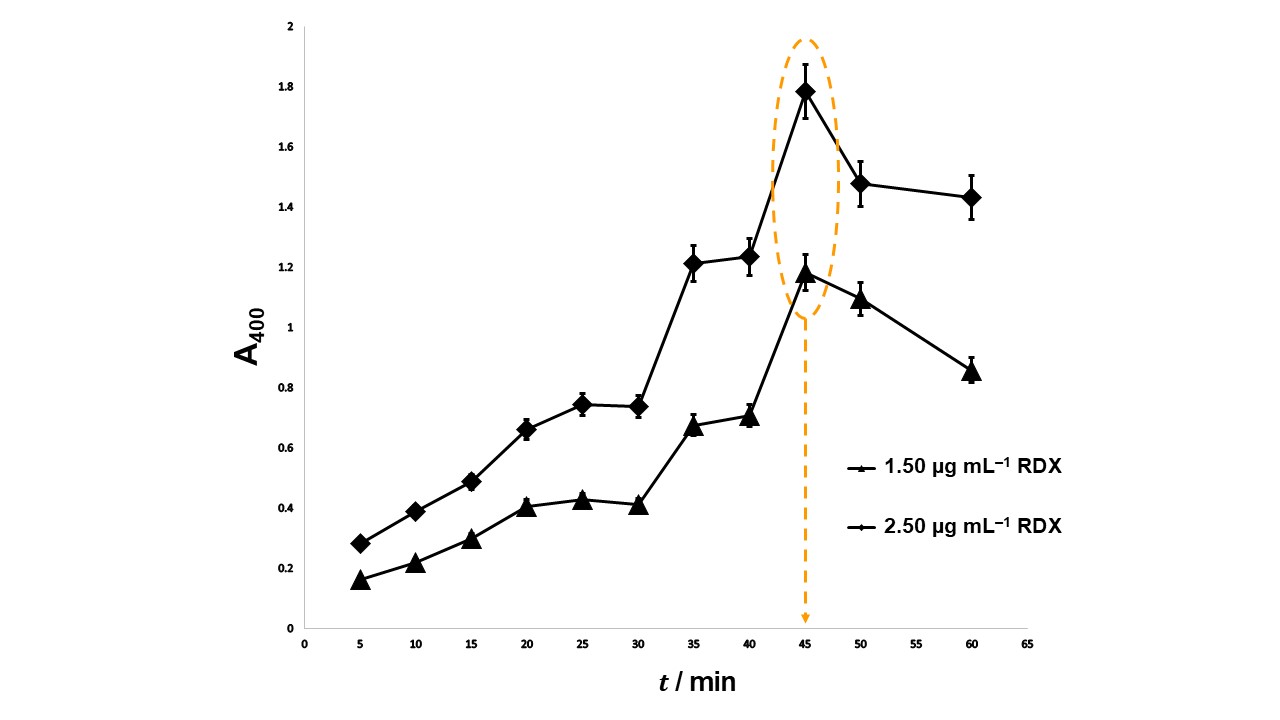


**Figure S5.** Optimization graph of absorbance values obtained for varying incubation times (5.0 – 60.0 minutes) by applying Ag(I)-diamine complex to 1.50 and 2.50 µg mL^-1^ (final concent.) RDX solutions {Exp. conditions: [AgNO_3_]_final_ = 2.16×10^–4^ mol L^–1^, [NaOH]_final_ = 6.4×10^–3^ mol L^–1^, [NH_3_]_final_ = 4.00×10^–3^ mol L^–1^, [RDX]_final_ = 10.0 µg mL^–1^, incubation temperature: 70 ^o^C}.

*
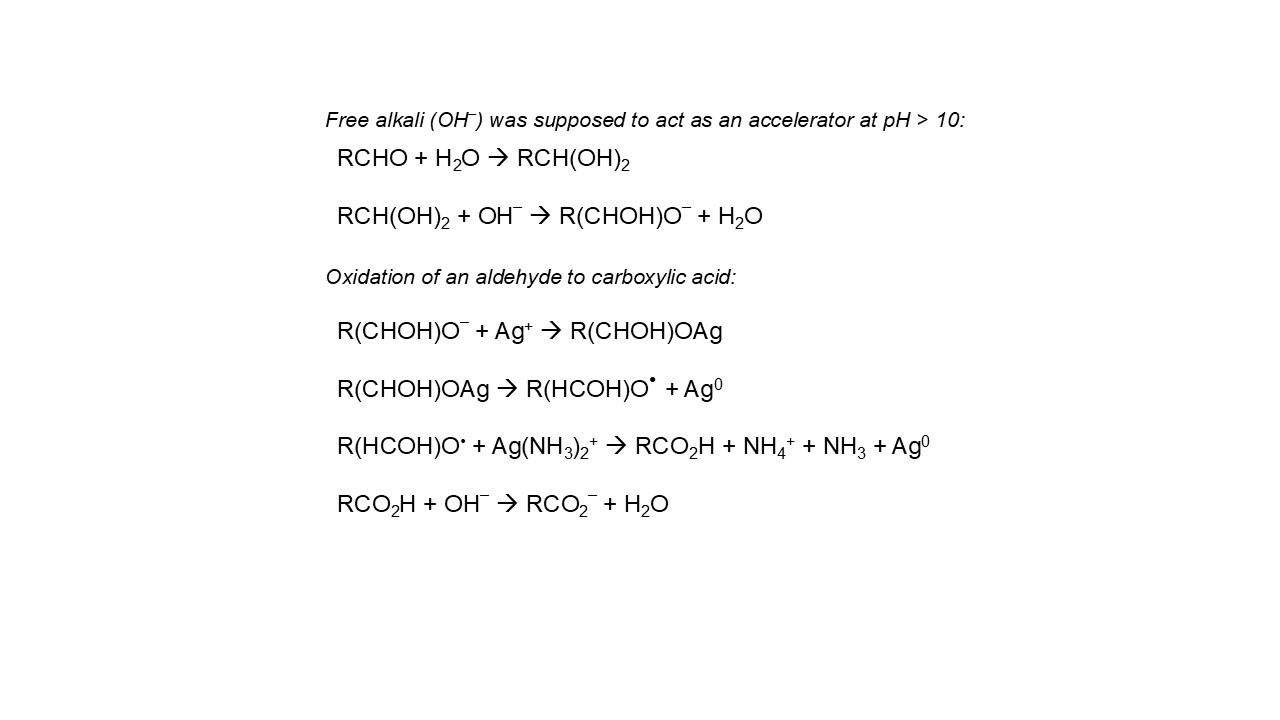
*

**Figure S6.** Reaction steps for the oxidation of an aldehyde to carboxylic acid by Ag^+^.


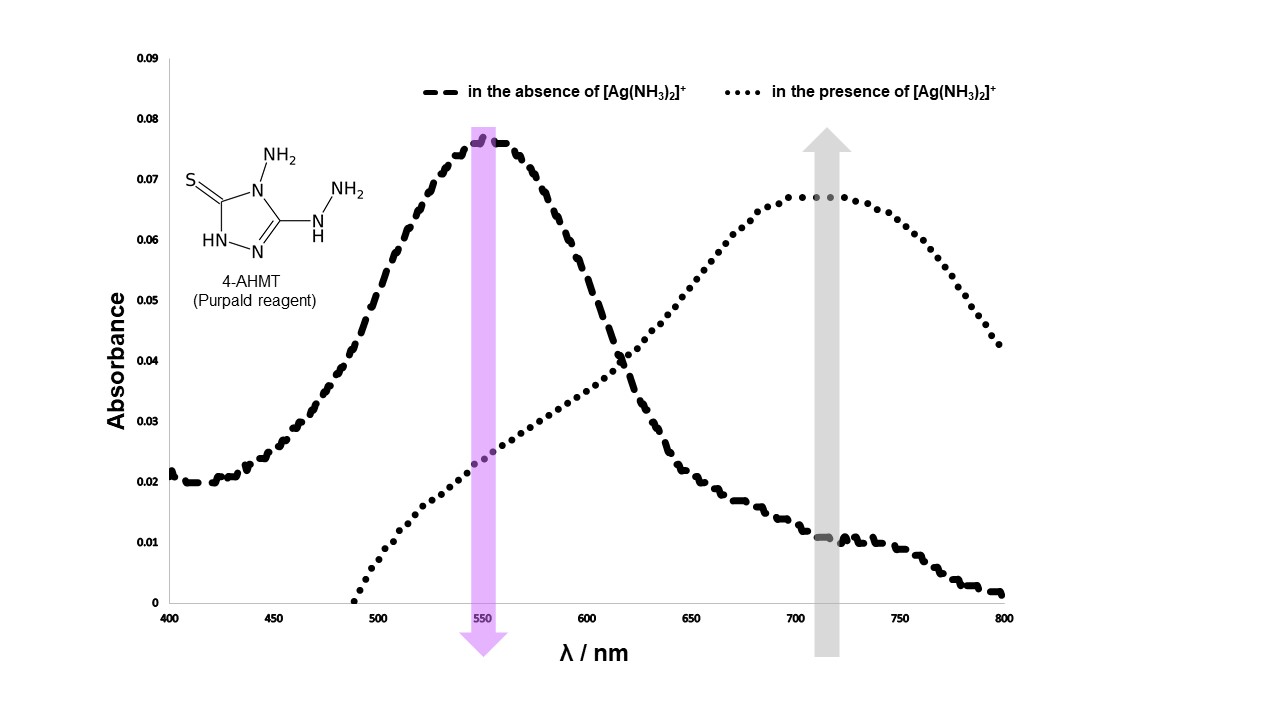


**Figure S7.** Visible spectra obtained by applying the Purpald^®^ test to RDX hydrolysates obtained in the absence and presence of [Ag(NH_3_)_2_]^+^ under the certain hydrolysis conditions of the proposed method (including chemical structure of Purpald reagent as inset figure).


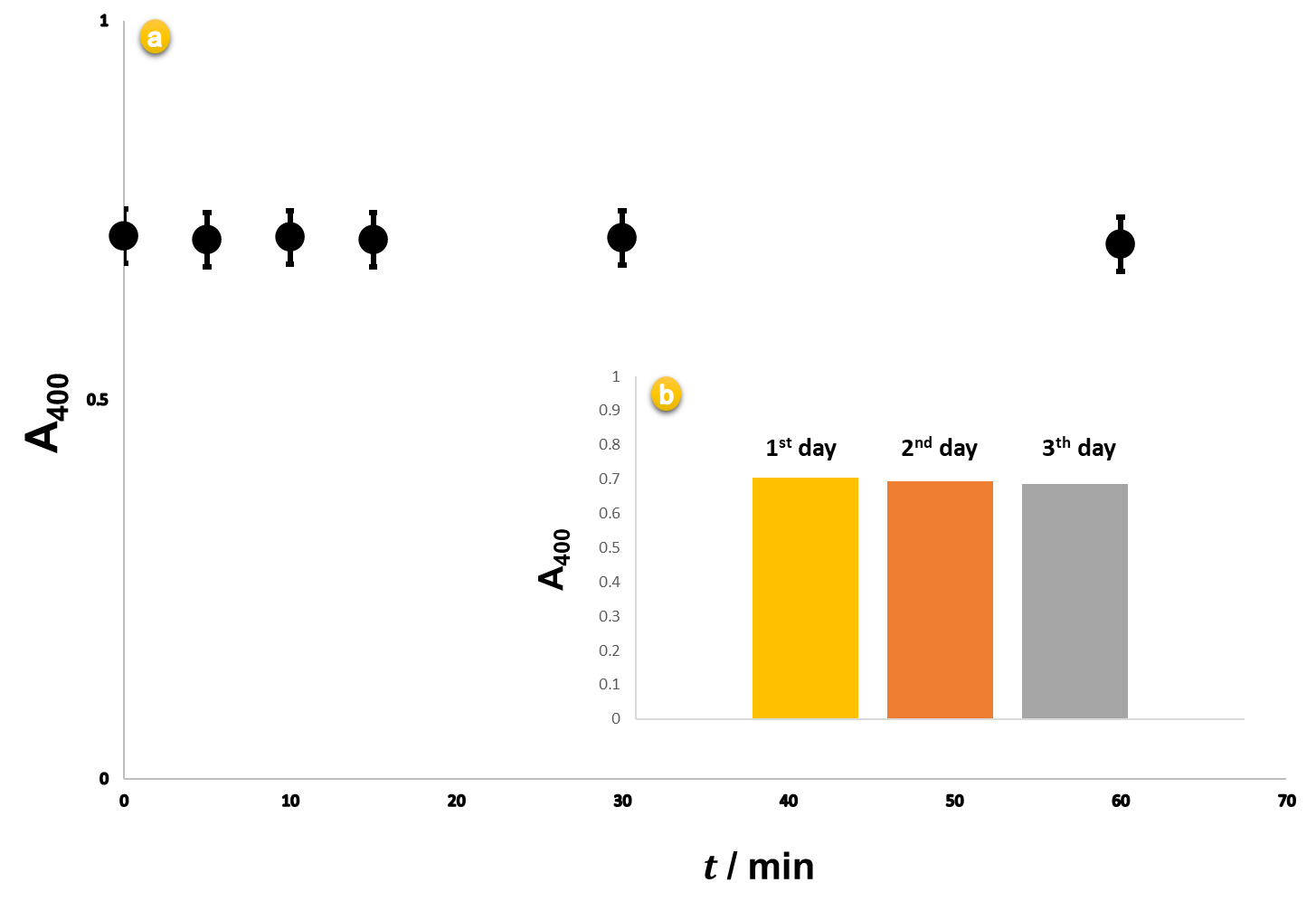


**Figure S8.** Absorbance values measured during 60-minute (a) and also 3 days (b) of the resulted AgNPs stored in room temperature.


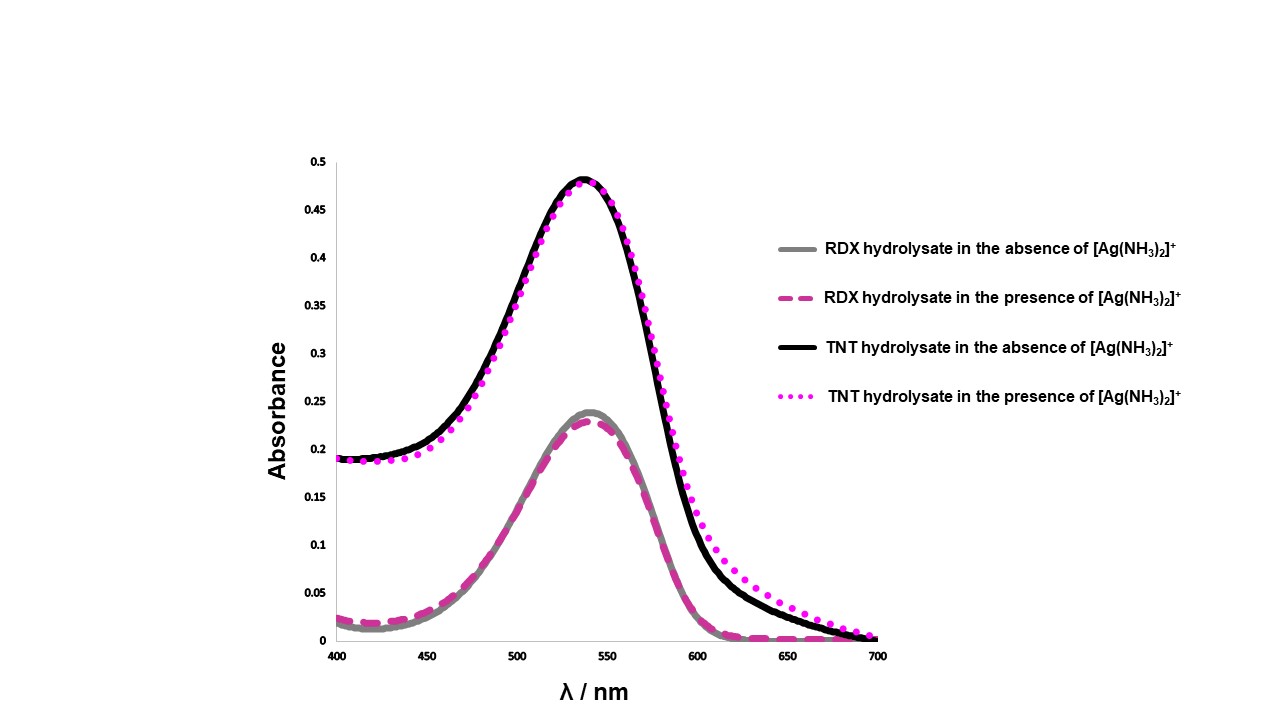


**Figure S9.** Visible spectra obtained by applying Griess test to RDX and TNT hydrolysates obtained in the absence and presence of [Ag(NH_3_)_2_]^+^ under hydrolysis conditions of the proposed method (TNT was used more concentrated because it releases lower amounts of nitrite than RDX in alkaline media^6^).


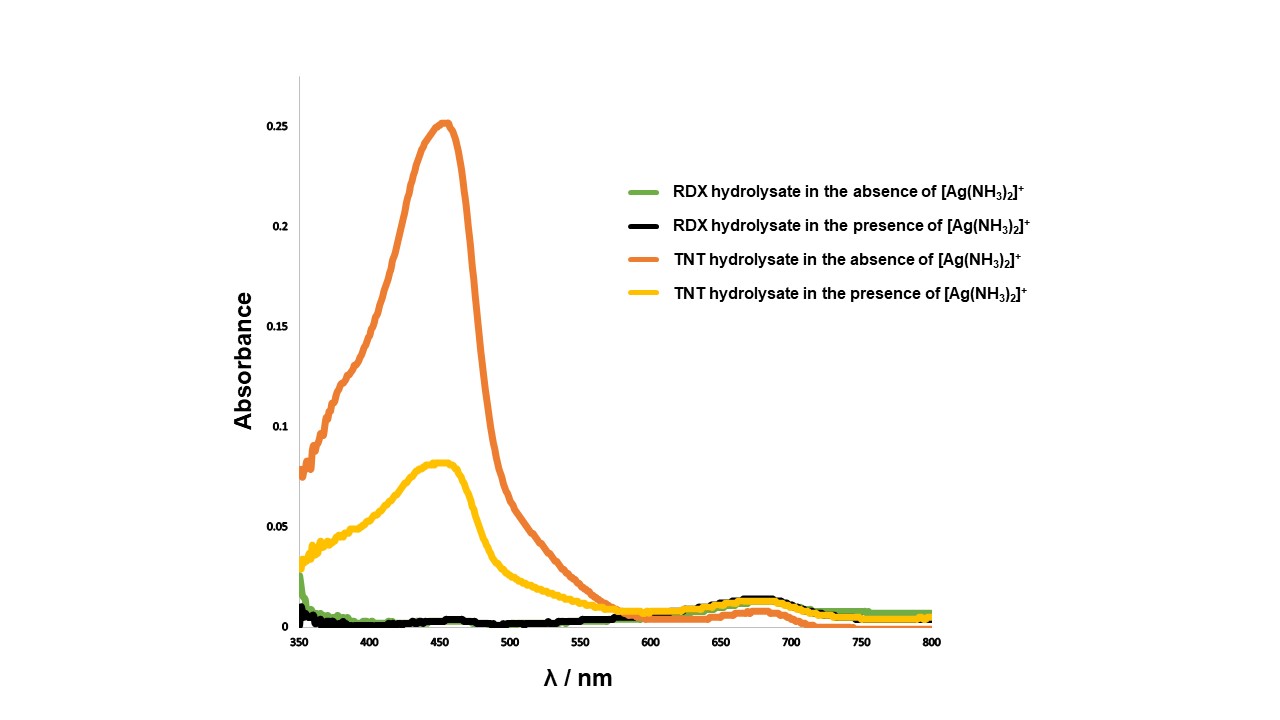


**Figure S10.** Visible spectra obtained by applying the CUPRAC test to RDX and TNT hydrolysates obtained in the absence and presence of [Ag(NH_3_)_2_]^+^ under the hydrolysis conditions of the proposed method.


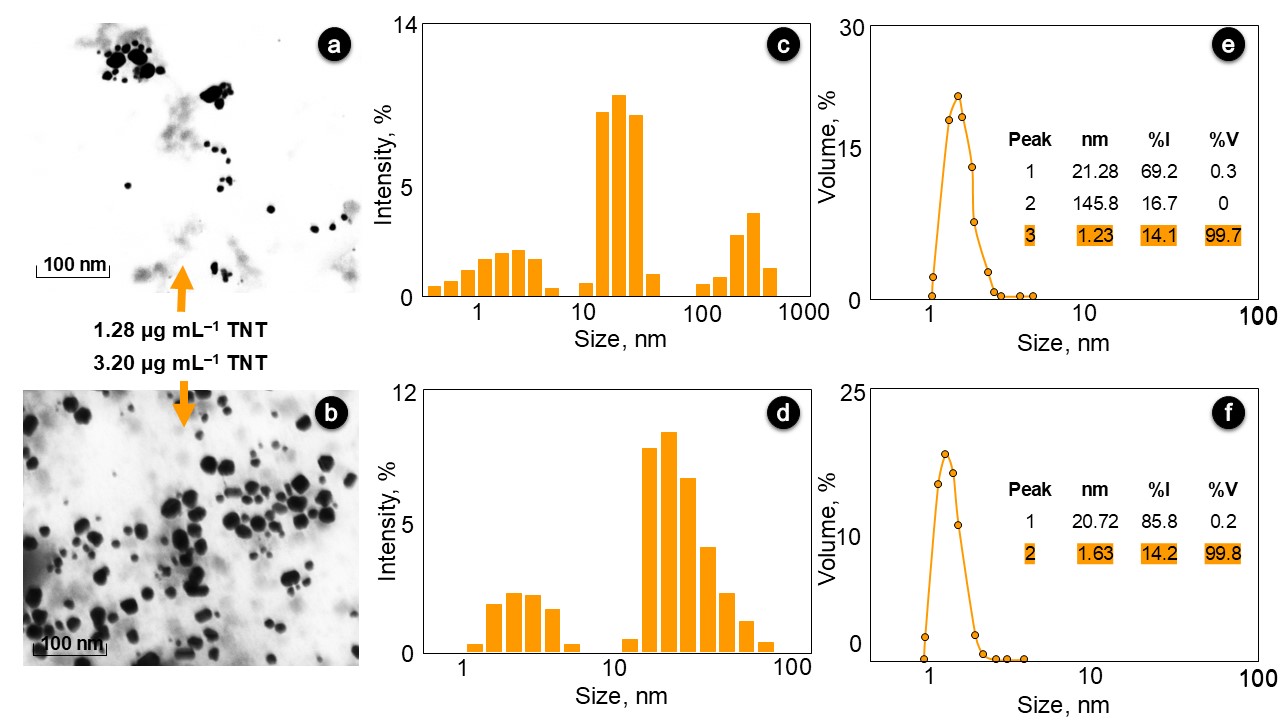


**Figure S11.** STEM images of *in situ* formed AgNPs using low (a) and high (b) conc. of TNT with their DLS derived intensity (c,d) and volume (e,f) distributions {Exp. conditions: [AgNO_3_]_final_ = 2.16×10^–4^ mol L^–1^, [NaOH]_final_ = 6.4×10^–3^ mol L^–1^, [NH_3_]_final_ = 4.00×10^–3^ mol L^–1^, incubation temperature and time: 70 ^o^C and 45-min}.


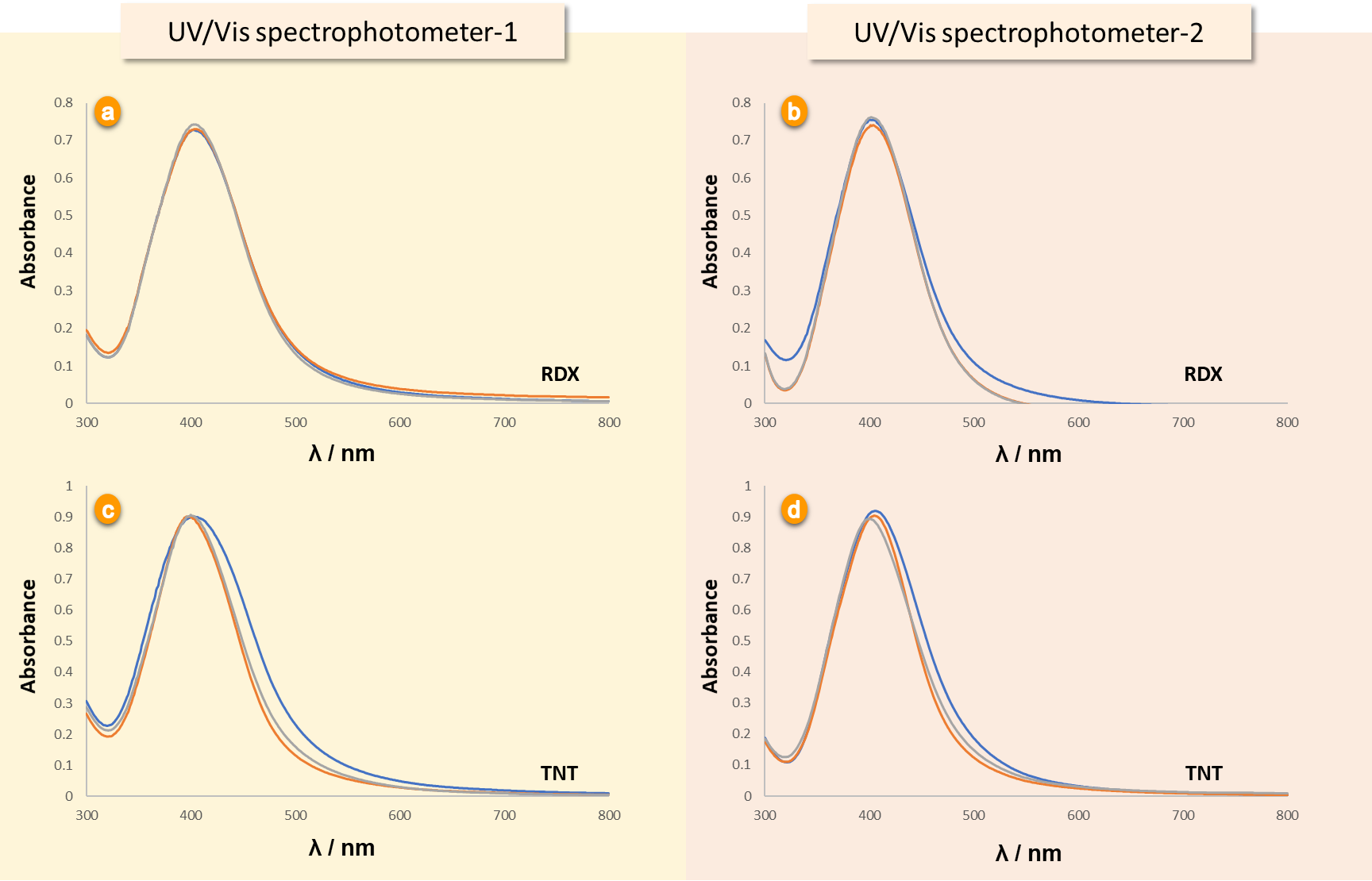


**Figure S11.** Visible spectra obtained using two different UV/Vis spectrophotometer-1 (Shimadzu UV–1900-i) and UV/Vis spectrophotometer-2 (Shimadzu UV–1800) after the application of the TR-driven system to RDX (a and b) and TNT (c and d) solutions of the same concentration in two different laboratories.


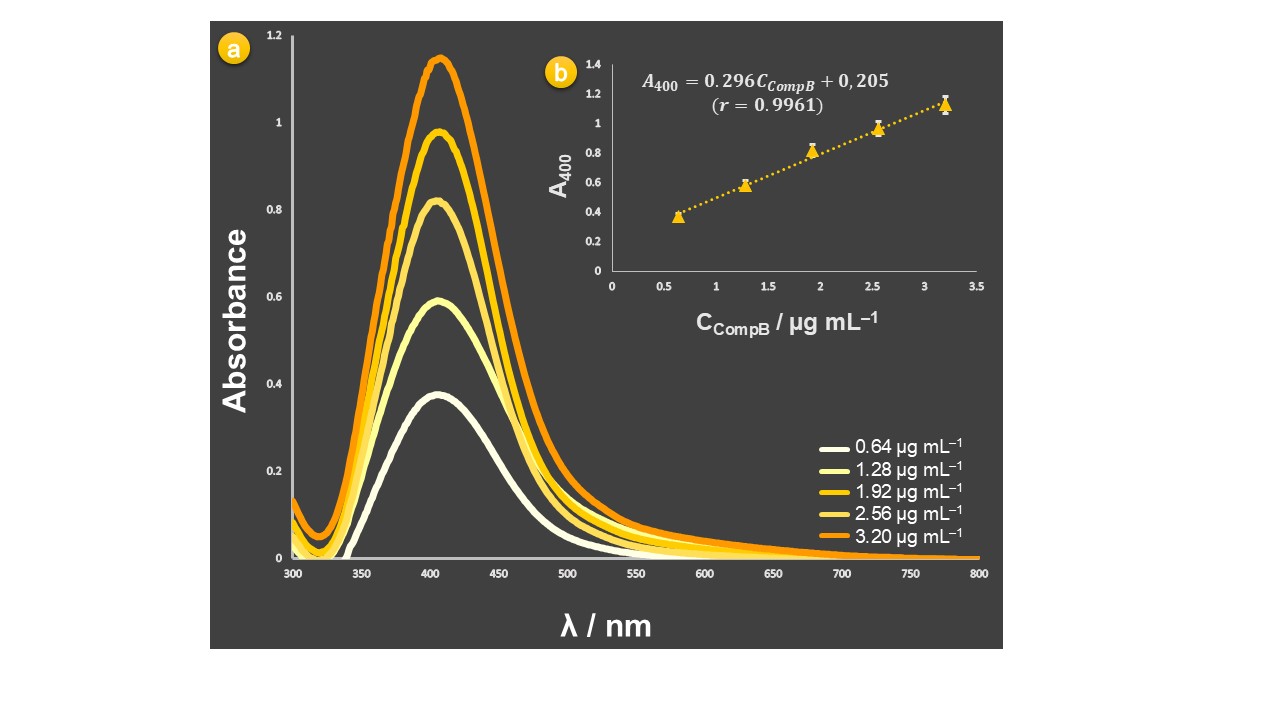


**Figure S13.** Visible spectra (a) and calibration curve (b) of in situ formed AgNPs applying of the proposed procedure to working solutions of Comp B in the final concentration range 0.64 – 3.2 µg mL^–1^ and blank sample solution (control) with image of the corresponding test tubes (as inset figure) from left to right containing blank sample solution (Comp B-free) and increasing concentration of Comp B {Exp. conditions: [AgNO_3_]_final_ = 2.16×10^–4^ mol L^–1^, [NaOH]_final_ = 6.4×10^–3^ mol L^–1^, [NH_3_]_final_ = 4.00×10^–3^ mol L^–1^, incubation temperature and time: 70 ^o^C and 45-min}.


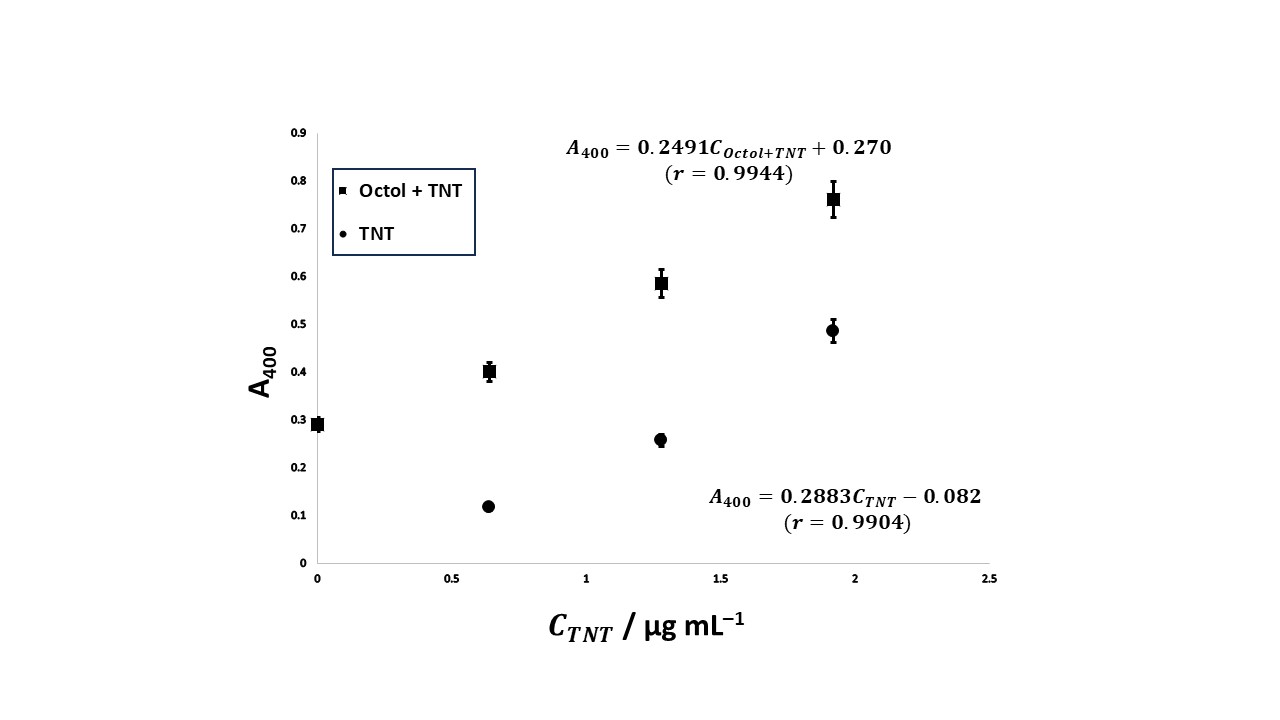


**Figure S14.** Comparison of calibration curves of TNT obtained by the proposed method alone and standard addition of TNT to 100.0 mg L^–1^ Octol.

# Supplementary Tables

**Table S1.** Results obtained on the recovery of RDX and TNT as a result of TNT separation by extraction process from soil samples.

| **RDX- and TNT-contaminated soil sample** | **A_found_** | **A_expected_** | **Recovery**  **(%)** |
| --- | --- | --- | --- |
| 320 µg mL^–1^ RDX (acetone extraction) | 0.449 | 0.422 | 106.4 ± 1.3 |
| 320 µg mL^–1^ TNT (toluene extraction) | 0.109 | 0.116 | 94.0 ± 0.7 |
| 640 µg mL^–1^ RDX:TNT (1:1, v/v)  (acetone extraction before toluene extraction) | 0.497 | 0.538 | 92.4 ± 0.5 |
| 640 µg mL^–1^ RDX:TNT (1:1, v/v) (after toluene extraction) – TNT | 0.129 | 0.116 | 111.2 ± 1.1 |
| 640 µg mL^–1^ RDX:TNT (1:1, v/v) (after acetone extraction) – RDX | 0.415 | 0.422 | 98.3 ± 1.7 |

**Table S2.** Recovery values (%) obtained by applying the proposed method to different types and mass ratios of explosive mixtures containing RDX.

| **Synthetic mixture** | **A_measured_** | **Recovery (%)** |
| --- | --- | --- |
| 0.96 µg mL^–1^ RDX | 0.456 | - |
| 9.60 µg mL^–1^ HMX | 0.092 | - |
| 9.60 µg mL^–1^ Tetryl | 0.016 | - |
| 9.60 µg mL^–1^ TNP | 0.001 | - |
| 9.60 µg mL^–1^ 2A-DNT | 0.014 | - |
| 9.60 µg mL^–1^ 4A-DNT | 0.012 | - |
| 9.60 µg mL^–1^ PETN | 0.042 | - |
| 0.96 µg mL^–1^ RDX + 9.60 µg mL^–1^ HMX | 0.469 | 102.8 ± 1.2 |
| 0.96 µg mL^–1^ RDX + 9.60 µg mL^–1^ Tetryl | 0.442 | 96.9 ± 0.9 |
| 0.96 µg mL^–1^ RDX + 9.60 µg mL^–1^ TNP | 0.448 | 98.2 ± 1.1 |
| 0.96 µg mL^–1^ RDX + 9.60 µg mL^–1^ 2A-DNT | 0.457 | 100.3 ± 0.7 |
| 0.96 µg mL^–1^ RDX + 9.60 µg mL^–1^ 4A-DNT | 0.447 | 98.1 ± 0.8 |
| 0.96 µg mL^–1^ RDX + 9.60 µg mL^–1^ PETN | 0.484 | 106.1 ± 3.2 |

**Table S3.** RDX recovery values obtained by investigating the interference effect of some ionic species and camouflage materials present in certain mass ratio along with RDX on the proposed method.

| **Interferent** | | **Recovery ± SD (%)** | **Tolerance ratio** |
| --- | --- | --- | --- |
| **Ionic**  **species** | Ca^2+^ | 103.1 ± 2.4 | ≥ 10 |
|  | Mg^2+^ | 100.7 ± 1.2 |  |
|  | Cu^II^* | 103.6 ± 0.9 |  |
|  | Fe^II^ | 101.8 ± 2.1 |  |
|  | Al^III^ | 93.7 ± 2.4 |  |
|  | SO_4_^2–^ | 99.0 ± 0.9 |  |
|  | Cl^–^ | 94.1 ± 1.1 |  |
|  | CO_3_^2–^ | 91.2 ± 0.8 |  |
| **Camouflage material** | Acetylsalicylic acid (aspirin) | 107.3 ± 5.2 | 10 |
|  | ᴅ-(+)-glucose* | 103.9 ± 1.2  108.4 ± 4.9 | 1  5 |
|  | Aspartame | 93.4 ± 2.5 | 10 |
|  | House-hold detergent | 98.2 ± 2.7 | 5 |

*interference effect of Cu^II^ and D-(+)-glucose to RDX determination was eliminated by solubility differences of the species in acetone as solvent.

**Table S4.** Recovery values obtained by applying the proposed method to synthetically prepared RDX and TNT mixtures at different mass ratios.

| $\boldsymbol{C}_{\boldsymbol{final}_{\boldsymbol{RDX}}}$ **:** $\boldsymbol{C}_{\boldsymbol{final}_{\boldsymbol{TNT}}}$ | **Mass ratio** | **A_measured_** | **A_expected_** | **Recovery (%)** |
| --- | --- | --- | --- | --- |
| 0.32 µg mL^–1^ RDX | **-** | 0.243 | **-** | **-** |
| 0.32 µg mL^–1^ TNT | **-** | 0.078 | **-** | **-** |
| 0.64 µg mL^–1^ RDX | **-** | 0.444 | **-** | **-** |
| 0.64 µg mL^–1^ TNT | **-** | 0.156 | **-** | **-** |
| 0.96 µg mL^–1^ RDX | **-** | 0.574 | **-** | **-** |
| 0.96 µg mL^–1^ TNT | **-** | 0.201 | **-** | **-** |
| 1.44 µg mL^–1^ RDX | **-** | 0.750 | **-** | **-** |
| 1.44 µg mL^–1^ TNT | **-** | 0.318 | **-** | **-** |
| 1.92 µg mL^–1^ RDX | **-** | 0.959 |  |  |
| 1.92 µg mL^–1^ TNT |  | 0.466 |  |  |
| 0.96 µg mL^–1^ RDX : 0.96 µg mL^–1^ TNT | 1:1 | 0.803 | 0.775 | 103.6 ± 0.9 |
| 0.96 µg mL^–1^ RDX : 1.92 µg mL^–1^ TNT | 1:2 | 1.039 | 1.04 | 99.9 ± 0.9 |
| 0.64 µg mL^–1^ RDX : 0.32 µg mL^–1^ TNT | 2:1 | 0.497 | 0.522 | 95.2 ± 1.4 |
| 0.32 µg mL^–1^ RDX : 0.96 µg mL^–1^ TNT | 1:3 | 0.414 | 0.444 | 93.2 ± 1.7 |
| 0.96 µg mL^–1^ RDX : 0.32 µg mL^–1^ TNT | 3:1 | 0.674 | 0.652 | 103.4 ± 0.9 |
| 0.96 µg mL^–1^ RDX : 1.44 µg mL^–1^ TNT | 2:3 | 0.903 | 0.892 | 101.2 ± 0.3 |
| 1.44 µg mL^–1^ RDX : 0.96 µg mL^–1^ TNT  (Mix ratio of Composite B) | 3:2 | 0.959 | 0.951 | 100.8 ± 0.2 |

**Table S5.** Recovery of TNT from synthetically prepared TNT-based munition mixtures.

| **Synthetic mixtures^a^ and their compositions^b^** | $\boldsymbol{C}_{\boldsymbol{final}_{\boldsymbol{TNT}}}$ | **A_measured_** | **A_expected_** | **Recovery (%)** |
| --- | --- | --- | --- | --- |
|  | 2.56 µg mL^–1^ TNT | 0.687 | 0.687 | **-** |
| 50.0 µg mL^–1^ Amatol  (20% TNT and 80% NH_4_NO_3_) | 2.56 µg mL^–1^ TNT | 0.678 |  | 98.70 ± 2.1 |
| 50.0 µg mL^–1^ Pentolit  (50% TNT and 50% PETN) | 2.56 µg mL^–1^ TNT | 0.706 |  | 102.8 ± 2.7 |

^a^under the name of the defense sector; ^b^as percentages by weight

**Table S6.** Statistical comparison at 95% confidence level of the reference LC–MS/MS method and the proposed spectrophotometric method for the analysis of RDX standards and RDX-contaminated soil samples (Expected C_RDX_: 32.0 µg mL^–1^, dilution factor: 6.4)

| **Sample** | **Parameter** | **Proposed**  **spectrophotometric method** |  | **Reference LC−MS/MS**  **method** |
| --- | --- | --- | --- | --- |
| RDX standards | Number of samples  Average  Standard deviation  Pooled standard deviation^a,b^  Degrees of freedom^a,b^  t_experimental_^a,b^  t_critical_^b^  F_experimental_^a,b^  F_critical_^b^ | 5  33.8  0.44 | 0.70  8  1.022  2.776  4.09  6.390 | 5  34.4  0.89 |
| RDX-contaminated soil | Number of samples  Average  Standard deviation  Pooled standard deviation^a,b^  Degrees of freedom^a,b^  t_experimental_^a,b^  t_critical_^b^  F_experimental_^a,b^  F_critical_^b^ | 3  34.17  0.29 | 0.285  4  1.94  4.303  1.07  19.0 | 3  32.3  0.28 |

^a^ S2 = ((n1 – 1)s12 + (n2 – 1)s22) / (n1 + n2 – 2) and t = (ā1 – ā2) / (S (1/n1 + 1/n2)1/2), where  s1 and s2 are the standard deviations of the two populations with sample sizes of n1 and n2, and sample means of ā1 and ā2 respectively (t has (n1 + n2 – 2) degrees of freedom); ^b^ Statistical comparison at 95% confidence level on paired data obtained with the proposed and reference methods (P = 0.05)

**Table S7.** Comparison of the pretreatment processes, detection mechanisms and analytical performance parameters of the proposed method with other reported colorimetric methods for RDX detection.

| **Pre-hydrolysis or degradation** | **Detection mechanism** | **LOD** | **Interference effect of TNT and removal process** | **Real sample** | **Reference** |
| --- | --- | --- | --- | --- | --- |
| Pretreatment with zinc powder and glacial acetic acid:  Nitrite formation | Formation of a reddish azo dye in the presence of the degradation product nitrite using Griess reagent  (λ_max_ = 507 nm) | - | Red-colored Meisenheimer complexation  (λ_max_ = 540 nm)  Removal with an anion exchange resin | RDX-contaminated soil samples | 7 |
| Zn + HCl reduction:  Formation of ammonia and amine | Formation of blue indophenol dye as a result of Berthelot reaction in alkaline medium (λ_max_= 631 nm) | 0.18 µg mL^–1^ | Removal by DCHA-IBMK extractive-spectrophotometric method | Analysis of Comp A5, Comp C4, Hexal and RDX-contaminated soil samples | 8 |
| Pre-hydrolysis with (NaOH + Na_2_CO_3_) followed by neutralization process:  Nitrite formation | The formation of purple azo dye as a result of the diazotization reaction of the released nitrite with 4-aminothiophenol modified AuNPs and N-(1-Naphthyl)ethylenediamine (λ_max_ = 565 nm) | 0.20 µg mL^–1^ | Elimination of low amount of nitrite released from TNT decomposition in alkaline environment by dilution effect | RDX-contaminated soil samples | 6 |
| Photolysis with  254 nm UV lamp:  Formation of formaldehyde | Formation of hydrozone by formaldehyde with hydrazine-substituted boron-dipyrromethene probe | 85.8 nmol L^–1^ | No interference effect | - | 9 |
| Nitrite formation by basic hydrolysis with NaOH | Inhibition of etching of Ag nanoprisms under alkaline conditions under natural sunlight by nitrite released from RDX as a result of alkaline hydrolysis: color change from pink (λ_max_ = 506 nm) to blue (λ_max_ = 686 nm) | 1 nmol L^–1^ | No interference effect | Natural water and soil samples | 10 |
| Simultaneous alkaline hydrolysis with NaOH and neutralization step | Redox reaction between HNO_2_ and TMB, which is formed as a result of the combination of two-phase Pickering NPs, one containing TMB in acidic medium and the other containing NaOH solution and RDX:  Formation of yellow-colored TMB-diimine radical cation  (λ_max_ = 450 nm) | 96 nmol L^–1^ | Not examined | RDX-contaminated lake and river water | 11 |
| In situ alkaline hydrolysis without the need for neutralization:  Formaldehyde formation | In situ AgNPs formation as a result of redox reaction between formaldehyde released by in situ alkaline hydrolysis of RDX and Ag(I)-diamine cationic complex exists in the Tollens' reagent-driven system  (λ_max_ = 400 nm) | 50.3 nmol L^–1^  (for RDX)  67.2 nmol L^–1^  (for TNT) | Linear response from TNT with AgNPs formation and simple extractive separation from soil samples with solubility difference in toluene | Analysis of Comp B, Comp A5, Octol and RDX-contaminated soil samples | This work |

# Supplementary References

1. Quesenberry, M. S.; Lee, Y. C. A Rapid Formaldehyde Assay Using Purpald Reagent: Application Under Periodation Conditions. *Anal. Biochem*. **1996**, *234*, 50–55.
2. Griess, P. Griess Reagent: A Solution of Sulphanilic Acid and α-naphthylamine in Acetic Acid which Gives a Pink Colour on Reaction with the Solution Obtained after Decomposition of Nitrosyl Complexes. *Chem. Ber.*, **1879**, *12*, 427.
3. Apak, R.; Güçlü, K.; Özyürek, M.; Karademir, S.E. Novel total antioxidant capacity index for dietary polyphenols and vitamins C and E, using their cupric ion reducing capability in the presence of neocuproine: CUPRAC method. *J. Agric. Food Chem.* **2004**, *52*, 7970–7981.
4. Şen. N.; Üzek, U.; Aksoy, Ç.; Bora, T.; Atakol, O. Farklı Yapıdaki Organik Patlayıcı Maddelerin LC-MS-MS ile Belirlenmesi. *Süleyman Demirel University Faculty of Arts and Science Journal of Science*, **2015**, *10*, 95–106. <https://doi.org/10.29233/sdufeffd.134823>
5. Sağlam, S.; Üzer, A.; Erçağ, E.; Apak, R. Electrochemical Determination of TNT, DNT, RDX, and HMX with Gold Nanoparticles/Poly(Carbazole-Aniline) Film–Modified Glassy Carbon Sensor Electrodes Imprinted for Molecular Recognition of Nitroaromatics and Nitramines. *Anal. Chem.* **2018**, *90*, 7364–7370.
6. Üzer, A.; Can, Z.; Akın, İ.; Erçağ, E.; Apak, R. 4-Aminothiophenol Functionalized Gold Nanoparticle-Based Colorimetric Sensor for the Determination of Nitramine Energetic Materials. *Anal. Chem.* **2014**, *86*, 351–356.
7. Üzer, A.; Erçağ, E.; Apak, R. Selective Colorimetric Determination of TNT Partitioned Between an Alkaline Solution and a Strongly Basic Dowex 1-X8 Anion Exchanger. *Forensic Sci. Int.* **2008**, *174*, 239–243.
8. Jenkins, T. F.; Walsh, M. E. 1992, Development of Field Screening Methods for Tnt, 2, 4-DNT and RDX in Soil. *Talanta*. **1992**, *39*, 419–428.
9. Gao, J.; Chen, X.; Chen, S.; Meng, H.; Wang, Y.; Li, C.; Feng, L. The BODIPY-Based Chemosensor for Fluorometric/Colorimetric Dual Channel Detection of RDX and PA. *Anal. Chem.* **2019**, *91*, 13675–13680.
10. He, Y.; Wang, L. Base-Driven Sunlight Oxidation of Silver Nanoprisms for Label-Free Visual Colorimetric Detection of Hexahydro-1,3,5-Trinitro-1,3,5-Triazine Explosive. *J. Hazard. Mater.* **2017**, *329*, 249–254.
11. Xie, Z.; Ge, H.; Du, J.; Duan, T.; Yang, G.; He, Y. Compartmentalizing Incompatible Tandem Reactions in Pickering Emulsions to Enable Visual Colorimetric Detection of Nitramine Explosives Using a Smartphone. *Anal. Chem.* **2018**, *90*, 11665–11670.
